# Wrinkled Strain‐Enriched High‐Entropy Metallene Enables Cross‐Site Tandem Nitrate‐to‐Ammonia

**DOI:** 10.1002/anie.5828704

**Published:** 2026-05-12

**Authors:** Tianfang Yang, Yang Liu, Menghao Kong, Shilong Li, Shizhe Liu, Guanjie He, Shuyan Gao

**Affiliations:** ^1^ School of Chemistry and Chemical Engineering Henan Normal University Xinxiang P. R. China; ^2^ School of Materials Science and Engineering Henan Normal University Xinxiang P. R. China; ^3^ Christopher Ingold Laboratory Department of Chemistry University College London London UK

**Keywords:** cross‐site tandem catalysis, electrochemical nitrate reduction reaction, high‐entropy metallene, lattice strain, Zn‐NO_3_^−^ battery

## Abstract

High‐entropy alloys (HEAs) are interesting for sustainable electrocatalytic nitrate‐to‐ammonia but suffer from low atom utilization and insufficient exposure of active sites. Here, we report a wrinkled PdFeCoNiCuIn high‐entropy metallene (HEM) that leverages a structural‐electronic dual regulation strategy to transcend these limitations. The incorporation of the p‐block element indium (In) triggers a strong p‐d orbital hybridization that synergizes with the intrinsic metallene architecture to construct a broad adsorption energy landscape. This architecture enables a cross‐site tandem catalytic pathway that spatially decouples nitrate activation from ammonia desorption, thereby circumventing scaling constraints. Consequently, the PdFeCoNiCuIn‐HEM delivers a remarkable ammonia Faradaic efficiency of 99.3% and a yield rate of 4.55 mmol h^−1^ mg_cat_^−1^. Furthermore, the rechargeable Zn‐NO_3_^−^ battery assembled with the PdFeCoNiCuIn‐HEM as cathode achieves a high open‐circuit voltage of 1.48 V, a power density of 7.36 mW cm^−2^, and stable cycling over 100 h. This work provides a practical and generalizable design strategy for developing efficient NRA catalysts.

## Graphical abstract

A wrinkled 2D PdFeCoNiCuIn high‐entropy metallene overcomes the low atom utilization of high‐entropy alloys. Indium‐induced strain and p‐d hybridization enable a cross‐site tandem pathway for nitrate reduction to ammonia. It achieves 99.3% Faradaic efficiency and an NH_3_ yield rate of 4.55 mmol h^−1^ mg_cat_^−1^. The PdFeCoNiCuIn‐HEM‐based Zn‐NO_3_^−^ battery delivers a peak power density of 7.36 mW cm^−2^ alongside 100‐h cycling stability.

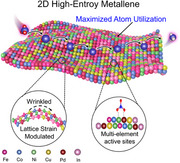

## 1 Introduction

Ammonia (NH_3_), with annual global demand exceeding 180 million tons, serves as a critical component in fertilizer manufacturing, chemical synthesis, and hydrogen‐rich fuel [1, 2]. NH_3_ is mostly synthesized using N_2_ and H_2_ by the energy‐intensive Haber–Bosch process, which accounts for approximately 2% of global energy consumption and contributes 1.4% of CO_2_ emissions, posing significant challenges to sustainable chemical production and climate mitigation efforts [3, 4, 5]. Meanwhile, excessive nitrate (NO_3_^−^) emissions from industrial effluents and agricultural runoff have driven ecological crises such as eutrophication [6]. Electrocatalytic nitrate reduction to ammonia (NRA) has attracted substantial attention as a green technology that converts nitrogen‐containing pollutants into value‐added ammonia fuel under ambient conditions [7, 8]. However, the coupled eight‐electron and nine‐proton transfer process is constrained by sluggish kinetics and rigid linear scaling relations (LSRs) [9]. Conventional mono‐ and bimetallic catalysts struggle to reconcile the conflicting thermodynamic requirements of strong NO_3_^−^ activation and facile NH_3_ desorption, making it extremely difficult to simultaneously achieve high catalytic activity and selectivity [10, 11].

**SCHEME 1:**
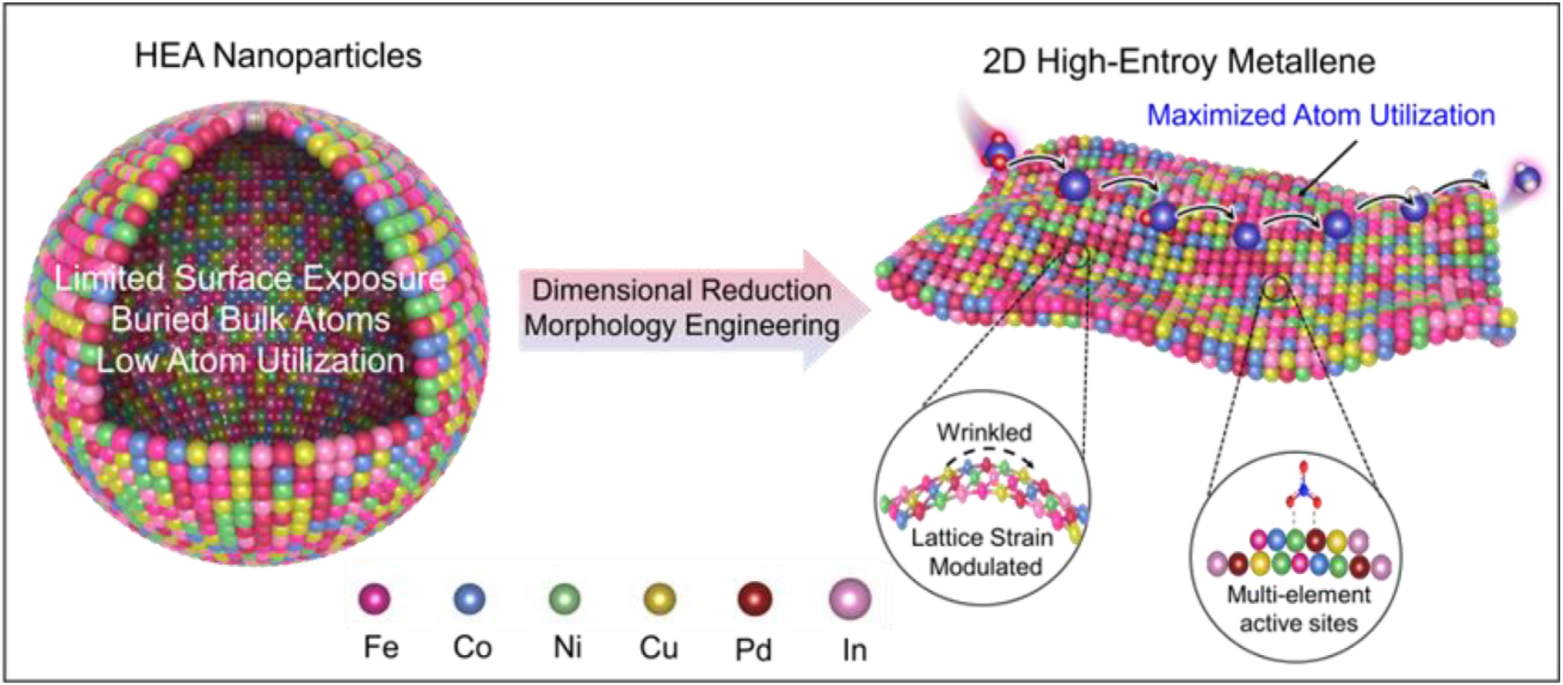
Schematic diagram of the design concept for a high‐entropy metallene catalyst for NRA.

High‐entropy alloys (HEAs) provide a novel strategy to enhancing the activity and selectivity in NRA, leveraging multi‐element synergistic effects and the tunable electronic structures induced by lattice distortion [12, 13, 14, 15]. However, the practical realization of this potential is severely hindered by the intrinsic crystallization habits of multi‐principal element systems. Driven by the tendency to minimize surface free energy and the requirement for high‐temperature synthesis to overcome positive mixing enthalpy, most conventional HEAs inevitably aggregate into thermodynamically stable nanoparticles or bulk phases during nucleation and growth [16, 17, 18]. In these dense solid morphologies, the vast majority of constituent atoms are buried deep within the bulk crystal lattice (Scheme 1), leading to critically low atom utilization efficiency and insufficient exposure of functional surface active sites [19]. Consequently, developing advanced structural engineering strategies to liberate these buried atoms and maximize the atomic utilization efficiency of HEAs remains a formidable challenge.

Herein, to address this intrinsic limitation, we propose the dimensional reduction of HEAs into two‐dimensional (2D) metallenes to maximize atomic utilization efficiency, reporting a structural‐electronic dual regulation strategy to synthesize a wrinkled PdFeCoNiCuIn high‐entropy metallene (HEM) (Scheme 1). In this design, the 3d transition metals (Fe, Co, Ni, and Cu) are selected as a fundamental matrix to facilitate initial NO_3_^−^ adsorption and stabilize various intermediates, while Pd is introduced to promote water dissociation and provide reactive active hydrogen for hydrogenation. The p‐block element In is incorporated to engineer a wrinkled, strained lattice while inducing p‐d orbital hybridization to globally weaken intermediate binding and facilitate NH_3_ desorption. Theoretical calculations and experimental results verify that the synergy of these diverse atomic sites enables a cross‐site tandem catalytic pathway that spatially decouples NO_3_^−^ activation from NH_3_ desorption and thereby circumvents scaling constraints. This work establishes a new paradigm for the rational design of complex reaction catalysts.

## 2 Results and Discussion

### 2.1 Preparation and Characterization of PdFeCoNiCuIn‐HEM

**FIGURE 1:**
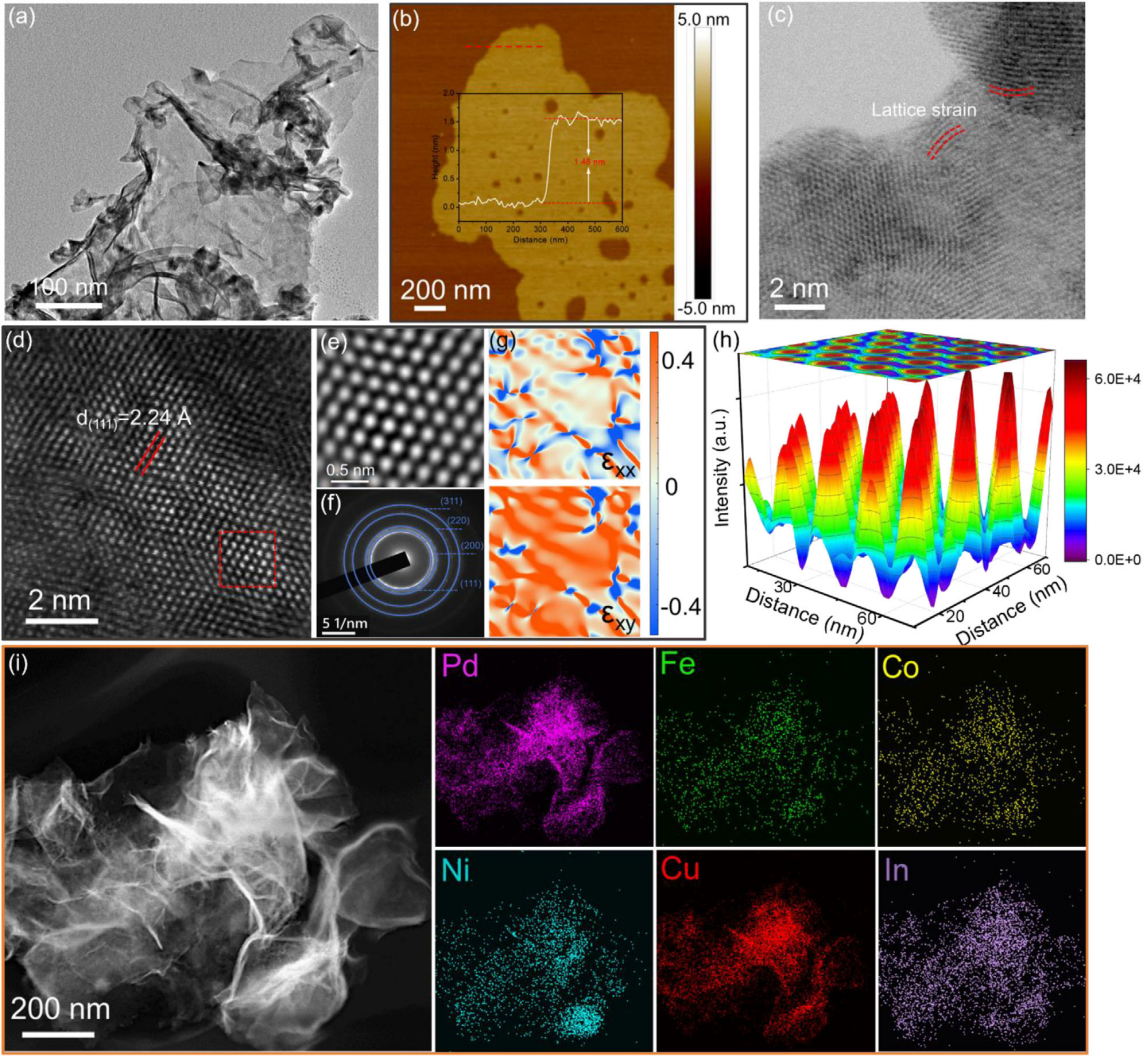
(a) TEM image, (b) AFM images, (c) ABF‐STEM image, (d) aberration‐corrected HAADF‐STEM image, (e) the inverse FFT (IFFT) patterns of the selected region in (d). (f) SAED pattern, (g) GPA images for strain distributions of *ε*_xx_ and *ε*_xy_ direction on (e) of PdFeCoNiCuIn‐HEM catalyst, the warm (orange) and cool (blue) colors in the strain contour plots display tensile strains and compressive strains with respect to the reference point, respectively. (h) 3D topographic images of atomic sites and relative intensity distribution, (i) HAADF‐STEM and EDS elemental mapping images of PdFeCoNiCuIn‐HEM.

A one‐step wet‐chemical route was developed to synthesize PdFeCoNiCuIn‐HEM. Scanning electron microscopy (SEM) and transmission electron microscopy (TEM) demonstrate a representative two‐dimensional ultrathin graphene‐like nanosheet structure rich in wrinkles and curves (Figures 1a and S3), which maximizes surface‐atom exposure and provides abundant accessible catalytic sites [20, 21]. The thickness of PdFeCoNiCuIn‐HEM is measured to be about 1.48 nm from the edge by atomic force microscopy (AFM) (Figure 1b). Annular bright‐field scanning TEM (ABF‐STEM) reveals pronounced lattice strains localized at the edge region, manifested by wrinkled and curved lattice fringes (Figure 1c). These strains are attributed to the intrinsically high curvature of the metallene and the concomitant multi‐element incorporation. Aberration‐corrected high‐angle annular dark‐field STEM resolves well‐defined lattice fringes with a spacing of 0.224 nm, indexed to the (111) plane (Figure 1d). Atomic‐resolution imaging shows a periodic lattice arrangement (Figure 1e), consistent with a face‐centered cubic (FCC) framework. The selected‐area electron diffraction (SAED) pattern displays sharp, indexable diffraction spots (Figure 1f), indicative of high crystallinity with pronounced crystallographic orientation.

Given the intrinsic atomic‐size mismatch among the constituent metals, geometric phase analysis (GPA) base on AC‐HAADF‐STEM is further performed to quantify local strain. Pronounced lattice strain is observed along ε_xx_ and ε_xy_ along the (111) and (200) crystal planes (Figure 1g), evidencing pervasive, entropy‐driven intrinsic tensile strain within the ultrathin lattice. Furthermore, three‐dimensional atomic‐site images and the corresponding compositional distribution maps clearly indicate intensity variations among atomic columns, indicative of configurational disorder and a high‐entropy elemental arrangement (Figure 1h). To verify atomic‐level mixing of the six constituents, high‐angle annular dark‐field scanning TEM (HAADF‐STEM) coupled with energy‐dispersive x‐ray spectroscopy (EDS) is employed. Elemental maps confirm a homogeneous distribution of Pd, Fe, Co, Ni, Cu, and In across the entire nanosheet without detectable phase segregation or compositional clustering (Figure 1i), corroborating the formation of a high‐entropy solid solution metallene. Furthermore, the quantitative element analysis (Table S1) confirms that the atomic ratios of the six elements are consistent with the design of a high‐entropy solid solution. The integration of these mapping results with the quantitative data verifies the compositional homogeneity of the PdFeCoNiCuIn‐HEM.

**FIGURE 2:**
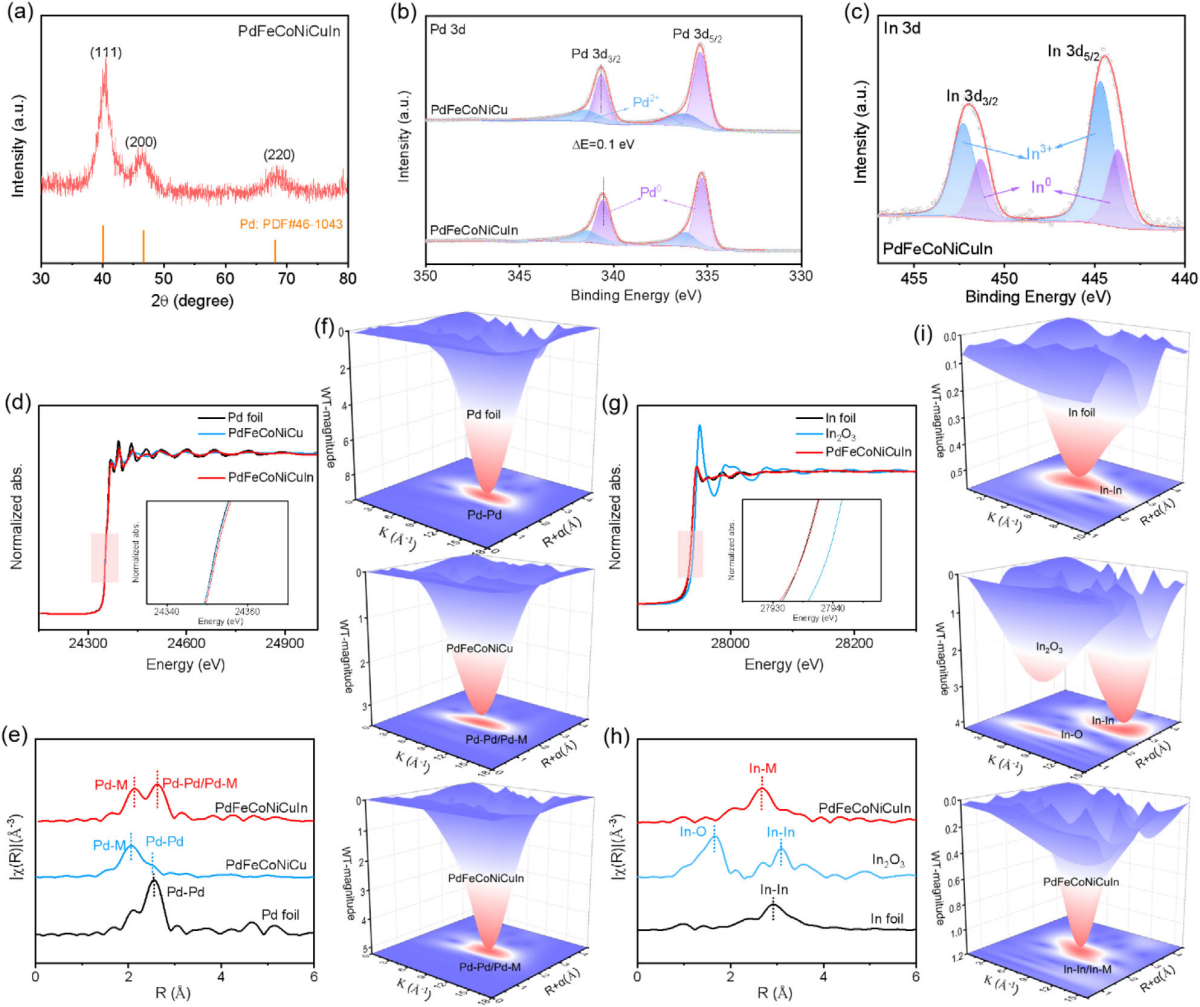
(a) XRD spectrum of PdFeCoNiCuIn‐HEM, orange dashed lines: PDF#46‐1043. (b) Pd 3d high‐resolution XPS spectra of PdFeCoNiCu and PdFeCoNiCuIn‐HEM. (c) In 3d high‐resolution XPS spectra of PdFeCoNiCuIn‐HEM. (d) XANES spectra, (e) FT k^2^‐weighted EXAFS spectra, (f) wavelet transform maps of PdFeCoNiCu and PdFeCoNiCuIn‐HEM at Pd K‐edge, the corresponding Pd foils serves as reference samples. (g) XANES spectra, (h) FT k^2^‐weighted EXAFS spectra, (i) wavelet transform maps of PdFeCoNiCuIn‐HEM at In K‐edge, the corresponding In foils and In_2_O_3_ serve as reference samples.

X‐ray diffraction (XRD) further manifests that PdFeCoNiCuIn‐HEM adopts a single‐phase FCC structure, aligning well with the TEM observations. The diffraction peaks at 40.3°, 46.4°, and 68.6° are indexed to the (111), (200), and (220) planes (PDF #46‐1043), respectively (Figure 2a). Compared with PdFeCoNiCu‐HEM (Figure S4), PdFeCoNiCuIn‐HEM shows obvious shifts of diffraction peaks towards lower angles, suggesting lattice expansion as a result of In intercalation. This pronounced macroscopic lattice expansion exceeds conventional solid‐solution expectations, arising from the severe atomic size mismatch of the incorporated In atoms and the extremely low out‐of‐plane structural constraint of the ∼1.48 nm 2D ultrathin metallene that completely releases internal lattice strain. Surface composition and chemical states are further examined by x‐ray photoelectron spectroscopy (XPS). Survey spectra (Figure S5) verify the presence of Pd, Fe, Co, Ni, Cu, and In. In the Pd 3d region, peaks at 335.3/340.5 eV and 336.1/341.4 eV correspond to Pd^0^and Pd^2+^ (3d_5/2_, 3d_3/2_) [22, 23], respectively (Figure 2b). The In 3d spectrum (Figure 2c) shows In^0^ at 443.7 and 451.3 eV [24], together with In^3+^ components at 444.7 and 452.3 eV [4]. Notably, the Pd 3d peaks shift slightly to lower binding energy relative to PdFeCoNiCu‐HEM, indicating electronic modulation and Pd electron enrichment induced by introducing p‐block In, consistent with their electronegativity difference (In 1.78 vs. Pd 2.20).

X‐ray absorption spectroscopy (XAS) displays pronounced heteroatomic coordination and charge redistribution in PdFeCoNiCuIn‐HEM. The normalized Pd K‐edge x‐ray absorption near‐edge structure (XANES) spectra of PdFeCoNiCuIn‐HEM display metallic edge features relative to the Pd foil (Figure 2d) [25]. The k^2^‐weighted Pd K‐edge x‐ray absorption fine structure (EXAFS) (Figure 2e) identifies two primary coordination shells. The first shell at ∼2.15 Å originates from heterogeneous Pd‐M bonds. Notably, the high‐coordination shell at ∼2.58 Å, predominantly Pd‐Pd in PdFeCoNiCu‐HEM, evolves into a broadened, mixed Pd‐Pd/Pd‐M shell in PdFeCoNiCuIn‐HEM. This significant spectral evolution, along with the overall reduction in amplitude, is driven by the heterogenization of the local coordination environment via Pd‐In interactions. Furthermore, the extreme size mismatch of In forced into the 2D ultrathin lattice induces intense local lattice distortion and strain, which directly modulates the bond lengths and increases the structural disorder around the Pd atoms. A smaller amplitude indicates the low coordinate environment of Pd atoms in PdFeCoNiCuIn‐HEM [26]. Consistently, wavelet‐transform (WT) analysis resolves an additional Pd‐M feature in PdFeCoNiCuIn‐HEM (Figure 2f), supporting a heterogeneous coordination environment and lattice distortion. At the In K‐edge, the absorption edge of PdFeCoNiCuIn‐HEM approximates that of In foil (Figure 2g) [27], indicating that In is predominantly metallic. The k^2^‐weighted In K‐edge EXAFS and WT analysis show a prominent peak at ∼2.69 Å assigned to In‐M coordination (Figure 2h,i), which is shorter than the In‐In bond in In foil (∼2.93 Å) by ∼0.24 Å, evidencing strong interatomic interactions.

### 2.2 Electrocatalytic Performance of NRA

**FIGURE 3:**
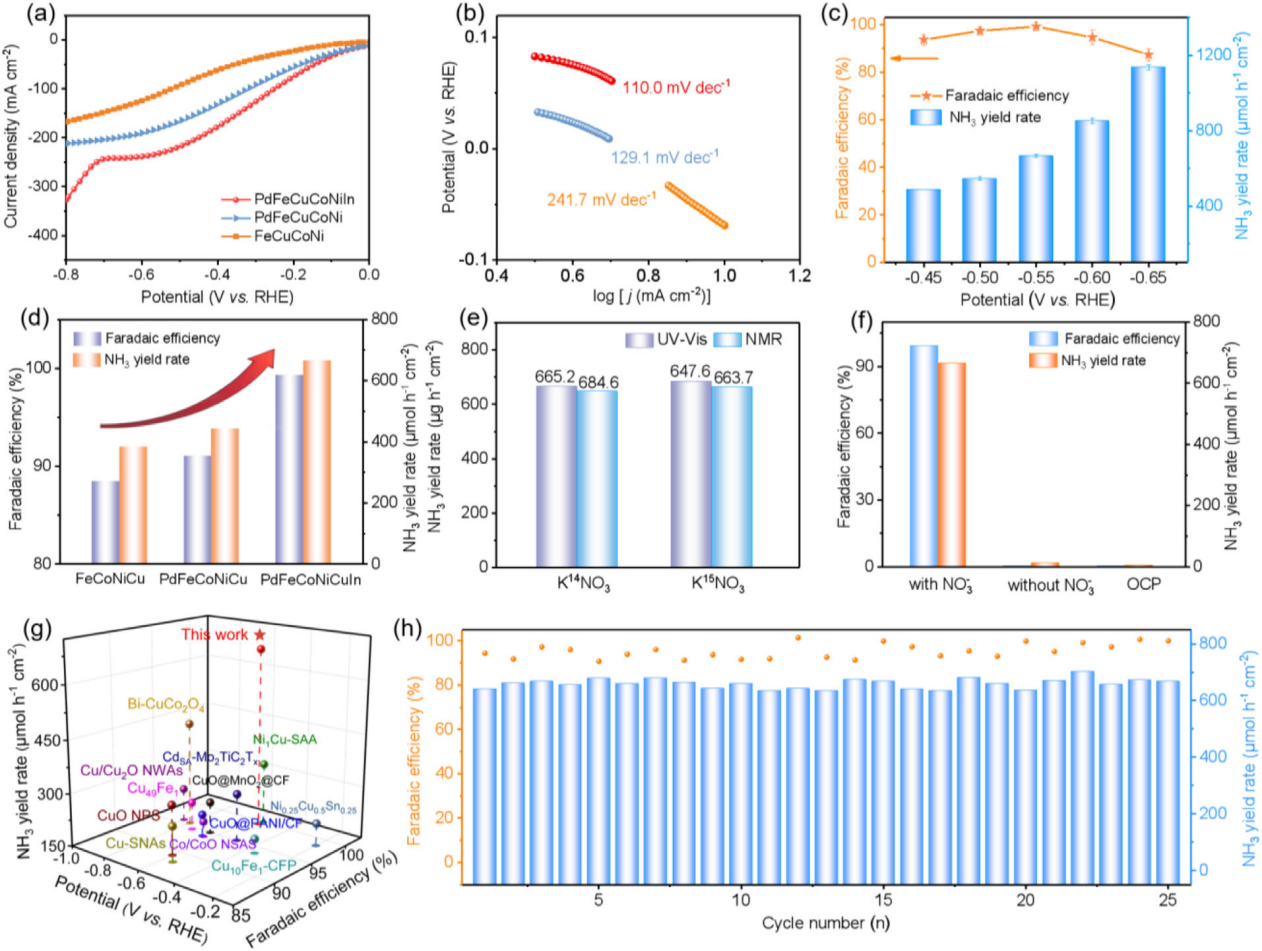
Electrocatalytic NRA performance. (a) LSV curves of FeCoNiCu‐MEA, PdFeCoNiCu ‐ HEM and PdFeCoNiCuIn‐HEM in 0.5 M K_2_SO_4_ and 0.1 M KNO_3_ solution. (b) Tafel slopes of FeCoNiCu‐MEA, PdFeCoNiCu, and PdFeCoNiCuIn‐HEM. (c) NH_3_ yield rate and FE of PdFeCoNiCuIn‐HEM at various potentials, error bars represent the standard deviation (SD; *n*  =  3, *n* derived from different experimental units), and data are presented as mean values ± SD. (d) NH_3_ yield rate and FE of NRA over different samples at −0.55 V. (e) NH_3_ yield rate and FE determined from UV‐Vis and ^1^H NMR methods. (f) NH_3_ yield rate in 0.5 M K_2_SO_4_ with/without NO_3_^−^, and at OCP with NO_3_^−^, respectively. (g) NH_3_ yield rate and FE comparison with other reported electrocatalysts. (h) Stability test at −0.55 V of PdFeCoNiCuIn‐HEM.

Catalyst composition strongly governs NRA activity and selectivity, with PdFeCoNiCuIn‐HEM showing superior overall performance. In 0.5 M K_2_SO_4_ + 0.1 M KNO_3_, linear sweep voltammetry (LSV) exhibits a pronounced NO_3_^−^‐induced current increase for PdFeCoNiCuIn‐HEM and consistently higher current densities than FeCoNiCu‐MEA and PdFeCoNiCu‐HEM (Figures 3a and S6). In addition, kinetic analysis further confirms that PdFeCoNiCuIn‐HEM delivers the smallest Tafel slope (110.0 mV dec^−1^) and the lowest charge‐transfer resistance (6.54 Ω) compared with the PdFeCoNiCu‐HEM and FeCoNiCu‐MEA (Figures 3b and S7, Table S2). The electrochemical active surface area (ECSA, Figure S8) reveals a significant increase for PdFeCoNiCuIn‐HEM (17.98 mF cm^−2^) relative to FeCoNiCu‐MEA (3.34 mF cm^−2^) and PdFeCoNiCu‐HEM (7.96 mF cm^−2^), evidencing enhanced active site exposure. Notably, PdFeCoNiCuIn‐HEM displays a volcano‐shaped NH_3_ Faradaic efficiency (FE) dependence from −0.45 to −0.65 V (vs. RHE), reaching 99.3% at −0.55 V versus RHE, while the NH_3_ yield rate maximizes at 1137.4 µmol h^−1^ cm^−2^ (4.55 mmol h^−1^ mg_cat_^−1^) at −0.65 V versus RHE (Figure 3c). At the optimal −0.55 V versus RHE (Figure 3d and Table S3), PdFeCoNiCuIn‐HEM delivers 99.3% FE and 665.3 µmol h^−1^ cm^−2^ NH_3_ yield rate, surpassing PdFeCoNiCu‐HEM (91.0%, 443.0 µmol h^−1^ cm^−2^) and FeCoNiCu‐MEA (88.5%, 383.0 µmol h^−1^ cm^−2^).

Isotope‐labeling experiments and blank experiments validate that NH_3_ derives from electrocatalytic NO_3_^−^ reduction. The ^1^H NMR spectra show the characteristic doublet of ^15^NH_4_^+^ and the distinct signal of ^14^NH_4_^+^ (Figure S9), ruling out extraneous nitrogen sources, and quantitative ^1^H NMR (calibration curves in Figure S2) agrees closely with UV‐vis analysis (Figure 3e), confirming the reliability and reproducibility of the measurements. Further, the absence of detectable NH_3_ in NO_3_^−^‐free electrolyte or at open‐circuit potential (OCP) (Figures 3f and S10) exhibits that the product originates solely from the electrocatalytic reduction process. Comparison of the electrocatalytic NRA performance of PdFeCoNiCuIn‐HEM for the NH_3_ yield rate and the FE of NH_3_ with that of previously reported catalysts (Figure 3g and Table S4) demonstrates its potential application in NH_3_ synthesis. Moreover, PdFeCoNiCuIn‐HEM also shows almost constant NH_3_ yield and FE during 25 cycles with fresh electrolytes (Figures 3h and S11), suggesting superior electrochemical stability for NRA. To further verify the structural robustness of the catalyst, physical characterizations are performed after the long‐term stability test (Figure S12). TEM images reveal that the original morphology is well‐preserved with negligible changes. Furthermore, the XRD pattern and XPS spectra demonstrate that the crystal structure and the chemical states of the constituent elements remain virtually unchanged compared to the initial sample. These results collectively prove the outstanding structural durability of PdFeCoNiCuIn‐HEM catalyst during the continuous electrocatalytic process.

### 2.3 Mechanistic Insights into Enhanced Catalytic NRA Activity

**FIGURE 4:**
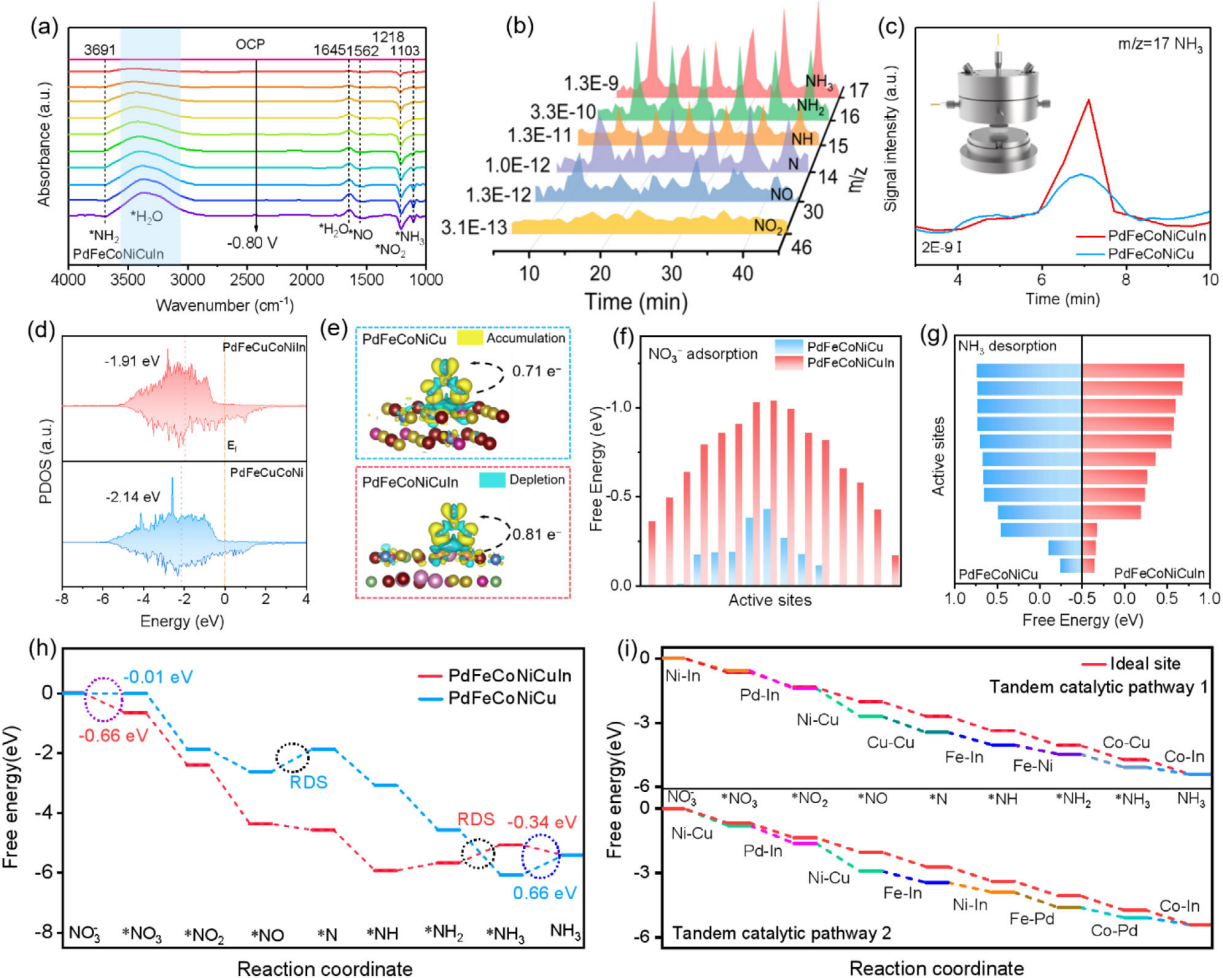
(a) In situ ATR‐FTIR spectra at different potential of PdFeCoNiCuIn‐HEM. (b) Online DEMS measurements during NRA on PdFeCoNiCuIn‐HEM. (c) Online DEMS measurements for detection NH_3_ on PdFeCoNiCuIn‐HEM and PdFeCoNiCu‐HEM. (d) PDOSs and d‐band centers of PdFeCoNiCuIn‐HEM and PdFeCoNiCu‐HEM, the abbreviation “E_f_” refers to “Fermi level.” (e) Charge density distributions of adsorbed NO_3_^−^ on PdFeCoNiCu‐HEM and PdFeCoNiCuIn‐HEM surfaces; the yellow and cyan contours indicate the charge accumulation and depletion, respectively. (f, g) Gibbs free energy diagrams of NO_3_^−^ adsorption and NH_3_ desorption on various sites of PdFeCoNiCuIn‐HEM and PdFeCoNiCu‐HEM. (h) Gibbs free energy diagrams for the NRA on PdFeCoNiCuIn‐HEM and PdFeCoNiCu‐HEM, the abbreviation “RDS” refers to “rate‐determining step.” (i) Tandem catalytic pathway with different combinations of active sites, the ideal active site refers to the uniform distribution of Gibbs free energy changes for the elementary reaction steps, with an average value of −0.67625 eV.

Given the pivotal role of active hydrogen (*H) as the immediate hydrogen donor for the hydrogenation of intermediates during NRA, reactive *H radicals are directly investigated via electron paramagnetic resonance (EPR) using DMPO as a spin‐trapping agent (Figure S13) [28, 29]. In NO_3_^−^‐free electrolyte, EPR spectra of PdFeCoNiCuIn‐HEM and PdFeCoNiCu‐HEM exhibit conspicuous DMPO‐H adduct signals (1:1:2:1:2:1:2:1:1) [30], implying the abundant formation of *H on their surfaces. In contrast, significantly weaker DMPO‐H signals are observed in the spectrum of FeCoNiCu‐MEA, demonstrating that the excellent *H supply capacity of PdFeCoNiCuIn‐HEM and PdFeCoNiCu‐HEM is attributed to the introduction of Pd sites. Upon introducing NO_3_^−^, the accumulated *H is implicated in the stepwise hydrogenation of N‐containing intermediates enroute to NH_3_. Next, in situ attenuated total reflection Fourier transform infrared (ATR‐FTIR) elucidates a stepwise NRA pathway on PdFeCoNiCuIn‐HEM (Figure 4a). While spectra feature no significant bands at OCP, a prominent *NO_2_ peak (1218 cm^−1^) [31] emerges at 0 V versus RHE and intensifies with cathodic potential. Concurrent signals at 1645 cm^−1^ (*H_2_O), 1562 cm^−1^ (*NO), and 3691 cm^−1^ (*NH_2_) [32] confirm water activation and sequential hydrogenation. At −0.80 V versus RHE, an *NH_3_ band appears at 1103 cm^−1^ [32, 33]. Notably, the superior intensity of *NH_3_ signal on PdFeCoNiCuIn‐HEM relative to PdFeCoNiCu‐HEM (Figure S14) suggests that In incorporation significantly promotes NH_3_ formation and desorption.

Complementary online differential electrochemical mass spectrometry (DEMS) establishes a selective, sequential hydrogenation pathway on PdFeCoNiCuIn‐HEM and the enhanced NH_3_ formation/desorption [34, 35]. During potential cycling from 0 to −0.9 V versus RHE, signals at m/z = 30 (NO), 17 (NH_3_), 16 (NH_2_), 15 (NH), and 14 (N) are detected (Figure 4b), consistent with stepwise hydrogenation along *NO_3_ → *NO_2_ → *NO → *N → *NH → *NH_2_ → *NH_3_ → NH_3_(g). In contrast, negligible signals at m/z = 2 (H_2_) and 28 (N_2_) are observed, supporting the high FE of NH_3_ (Figure S15). Notably, PdFeCoNiCuIn‐HEM exhibits a stronger NH_3_ signal than PdFeCoNiCu‐HEM (Figures 4c and S16), indicating that In incorporation favors NH_3_ production and release.

Density functional theory (DFT) calculations are employed to unravel the electronic origin of the enhanced catalytic performance and to elucidate the reaction mechanism on the high‐entropy surface. To understand the electronic modulation induced by the p‐block element, we first analyze the projected density of states (PDOS) and d‐band centers (*ε*_d_). As shown in Figure S17, the incorporation of In distinctly modulates the valence‐band structure compared to the quinary PdFeCoNiCu‐HEM. Specifically, the In 5p orbitals exhibit a broad energy overlap and distinct resonance with the 3d/4d orbitals of the entire transition metal matrix (in the range of ∼ −5.0 to 0 eV), providing direct evidence for a robust, collective p‐d orbital hybridization [36, 37]. Together with the lattice strain imposed by atomic‐size mismatch (consistent with GPA), this matrix‐wide electronic hybridization synergistically upshifts the overall d‐band center towards the Fermi level (E_f_), moving from −2.14 eV in the quinary system to −1.91 eV in the senary PdFeCoNiCuIn‐HEM (Figure 4d). Furthermore, the charge density distributions are visualized to illustrate the interaction between *NO_3_ and the catalyst surface (Figures 4e and S18). Notably, the charge transfer numbers of PdFeCoNiCuIn‐HEM to NO_3_^−^ (0.81 eV) donates more than PdFeCoNiCu‐HEM (0.71 eV), indicating that NO_3_^−^ can be effectively activated and polarized by PdFeCoNiCuIn‐HEM.

To further assess the thermodynamics of the initial NO_3_^−^ capture and final NH_3_ release, the Gibbs free energies of NO_3_^−^ adsorption and NH_3_ desorption over diverse active sites are statistically analyzed. Compared to the quinary PdFeCoNiCu‐HEM (Figure 4f and Table S5), the PdFeCoNiCuIn‐HEM exhibits a markedly broadened and more continuous adsorption‐energy distribution for *NO_3_, thereby increasing the population of near‐optimal adsorption configurations. Importantly, efficient catalysis requires both effective reactant activation and facile NH_3_ removal to avoid surface poisoning. Figure 4g and Table S6 show that In species incorporation lowers the Gibbs free energy of NH_3_ desorption relative to the quinary PdFeCoNiCu‐HEM. This observation suggests that the senary high‐entropy lattice offers a diverse continuum of capture sites, while In‐induced p‐d hybridization weakens NH_3_ binding to couple robust nitrate capture with facile ammonia release. Also, the Gibbs free energy landscape elucidates the kinetic superiority of the PdFeCoNiCuIn‐HEM (Figure 4h). The energy barrier of the rate‐determining step (RDS) for PdFeCoNiCuIn‐HEM (*NH_2_ → *NH_3_) is confirmed by free energy analysis to be lower than that of the RDS for PdFeCoNiCu‐HEM (*NO → *N), indicating that PdFeCoNiCuIn‐HEM is more favorable for NRA. Crucially, the In‐induced microstrain effect and p‐d orbital hybridization synergistically optimize the electronic structure to facilitate NO_3_^−^ adsorption while simultaneously weakening the binding strength of NH_3_, thereby ensuring swift product desorption and mitigating surface poisoning.

Finally, to elucidate the complex multistep mechanism on this highly heterogeneous surface, we depict the reaction kinetics using a “cross‐site tandem catalytic pathway” model (Figure 4i). The thermodynamic combinatorial analysis unveils a massive network where every elementary step is spontaneous (Δ*G* < 0). Although pathway 1 (Ni‐In → Pd‐In → Ni‐Cu → Cu‐Cu → Fe‐In → Fe‐Ni → Co‐Cu → Co‐In) exhibits the mathematically optimal trajectory with the smoothest energy landscape and the minimized mean squared error (MSE = 0.078) relative to the ideal linear decrease (Table S7), pathway 2 (Ni‐Cu → Pd‐In → Ni‐Cu → Fe‐In → Ni‐In → Fe‐Pd → Co‐Pd → Co‐In) clarifies a mechanistically superior alternative that perfectly matches the “strong capture‐facile release” criterion (Figure S19 and Table S8). Specifically, pathway 2 initiates with strong NO_3_^−^ activation (Δ*G* = −0.79 eV) on Ni‐Cu sites, proceeds through stable Fe‐mediated intermediates, and concludes with facile desorption at Co‐In sites (Δ*G* = −0.34 eV). The above results indicate that In‐induced strain and p‐d orbital hybridization orchestrate a cross‐site tandem pathway featuring “strong capture‐facile release”, thereby circumventing linear scaling constraints and balancing efficient NO_3_^−^ activation with facile NH_3_ desorption to enable sustained, high‐efficiency catalysis.

### 2.4 Zn‐NO_3_^−^ Battery Assembly and Evaluation

**FIGURE 5:**
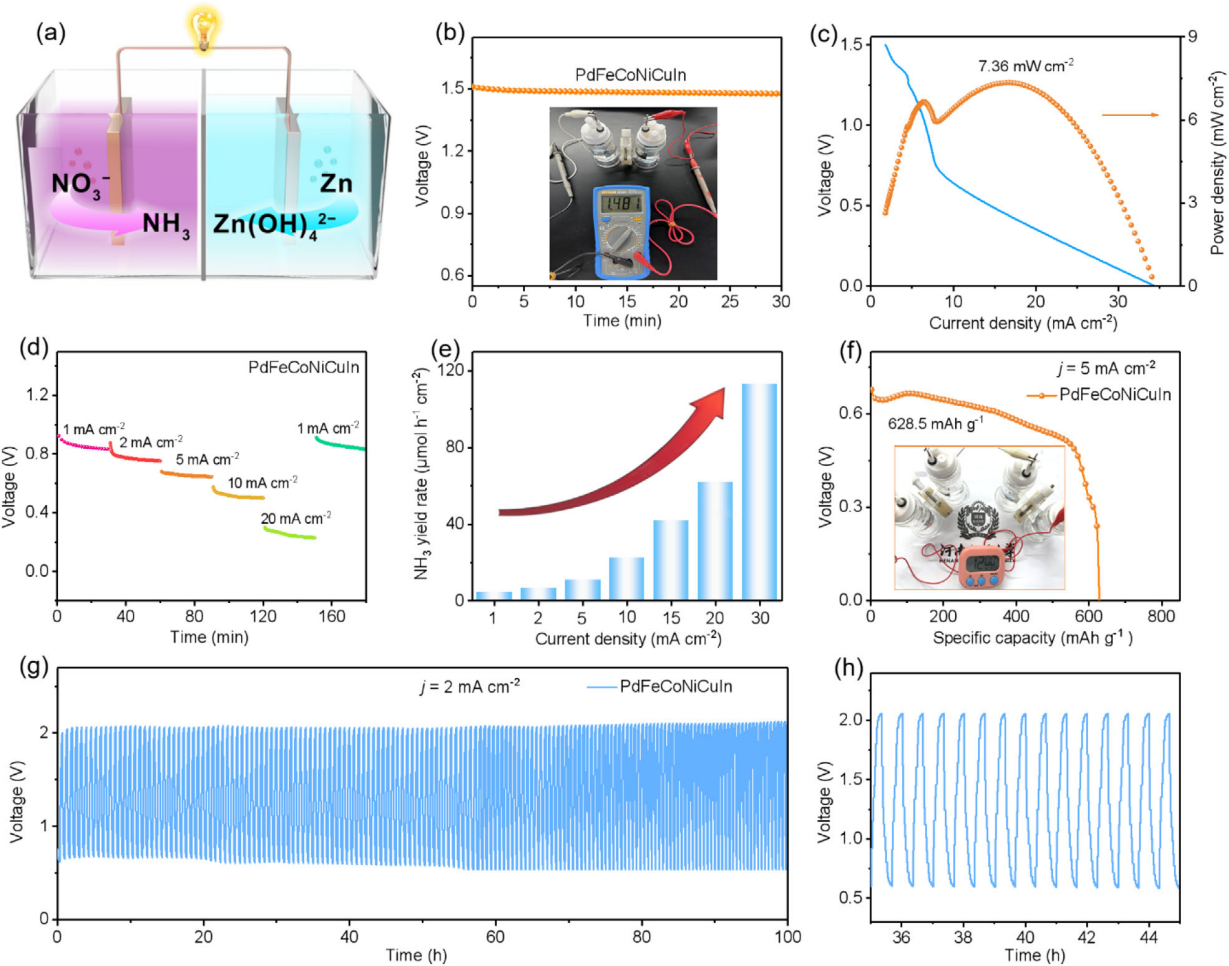
Zn‐NO_3_^−^ battery performance. (a) Schematic illustration of the PdFeCoNiCuIn‐based Zn‐NO_3_^−^ battery. (b) Open‐circuit voltage. (c) Discharge polarization and corresponding power density curves. (d) Galvanostatic discharge profiles at different current densities and corresponding (e) NH_3_ yield rate. (f) The specific capacity curves are normalized by the mass of the consumed Zn anode at 5 mA cm^−2^ (inset is the power supply of an electronic timer by two Zn‐NO_3_^−^ batteries in series). (g) Cyclic stability at a current density of 2 mA cm^−2^. (h) Representative partial charge‐discharge profiles at 2 mA cm^−2^ for the PdFeCoNiCuIn‐based Zn‐NO_3_^−^ battery.

Building on the outstanding NRA activity of PdFeCoNiCuIn‐HEM, we develop a Zn‐NO_3_^−^ battery that simultaneously enables sustainable NH_3_ production, efficient power output, and nitrate remediation (Figure 5a) [38, 39]. The PdFeCoNiCuIn‐HEM cathode and Zn‐foil anode operate in 0.5 M K_2_SO_4_ + 0.1 M KNO_3_ (catholyte) and 6 M KOH + 0.2 M Zn(Ac)_2_ (anolyte), respectively [40, 41]. The assembled cell delivers a stable open‐circuit voltage of 1.48 V (vs. Zn^2+^/Zn) (Figure 5b). Figure 5c shows that the typical discharging polarization and power density curves of the Zn‐NO_3_^−^ battery exhibit a high peak power density of 7.36 mW cm^−2^ at an optimal current density of 16.72 mA cm^−2^. We further evaluated the practical potential of PdFeCoNiCuIn‐HEM catalyst with higher mass loadings (2.0 and 5.0 mg cm^−2^). As the mass loading increases, the peak power density is further enhanced, reaching 9.12 mW cm^−2^ at a high loading of 5.0 mg cm^−2^ (Figure S20). As shown by the discharge polarization behavior (Figure 5d), the discharge plateau decreases with increasing current density, yet the voltage response recovers reversibly when the current density is returned to 1 mA cm^−2^, indicating excellent rate capability and discharge stability [42, 43]. Meanwhile, the NH_3_ yield rate increases monotonically with current density, reaching 451.2 µmol h^−1^ mg_cat_^−1^ at 30 mA cm^−2^ (Figure 5e). The PdFeCoNiCuIn‐HEM‐based Zn‐NO_3_^−^ battery delivers a high specific capacity of 628.5 mAh g^−1^, indicating strong energy‐storage capability (Figure 5f). Two Zn‐NO_3_^−^ batteries in series power an electronic timer (insets of Figure 5f), confirming the practical viability for energy supply applications. Furthermore, the battery maintains stable performance over 100 h of continuous galvanostatic discharge‐charge cycling at 2 mA cm^−2^, demonstrating long‐term durability and good rechargeability (Figure 5g,h). Notably, the practical and generalizable design is further validated by a successful gram‐scale synthesis with its high‐entropy structure confirmed by XRD (Figure S21), alongside a scaled‐up 10 × 10 cm^2^ battery delivering stable operation at 0.1 A for 60 h (Figure S22). Therefore, the PdFeCoNiCuIn‐HEM‐based Zn‐NO_3_^−^ battery exhibits remarkable power output, NH_3_ production and charge‐discharge stability, surpassing the performance of Zn‐NO_3_^−^ batteries reported in previous studies (Table S9).

## 3 Conclusions

In summary, we report the construction of a wrinkled PdFeCoNiCuIn‐HEM that integrates maximized atom utilization with intrinsic multi‐element synergistic effects. The utilization of the p‐block element In leverages size mismatch to engineer a wrinkled, strained lattice and induces p‐d orbital hybridization, thereby constructing a robust cross‐site tandem catalytic pathway. Consequently, the PdFeCoNiCuIn‐HEM delivers a remarkable NH_3_ FE of 99.3% and a yield rate of 4.55 mmol h^−1^ mg_cat_^−1^. Furthermore, the catalyst demonstrates exceptional practicality in a rechargeable Zn‐NO_3_^−^ battery, achieving a high open‐circuit voltage of 1.48 V, a power density of 7.36 mW cm^−2^, and stable cycling over 100 h. Ultimately, this work establishes a generalizable paradigm for designing efficient electrocatalysts by geometric morphology and harmonizing element synergy.

## Author Contributions

**Tianfang Yang**: investigation, writing – original draft, writing – review and editing, conceptualization, data curation. **Yang Liu**: methodology, funding acquisition, writing – review and editing. **Menghao Kong**: methodology, validation. **Shilong Li**: software, validation. **Shizhe Liu**: methodology, formal analysis. **Guanjie He**: funding acquisition, writing – review and editing, visualization. **Shuyan Gao**: funding acquisition, project administration, writing – review and editing, supervision, resources, conceptualization.

## Conflicts of Interest

The authors declare no conflicts of interest.

## Supporting information




**Supporting File**: anie72585‐sup‐0001‐SuppMat.docx.

## Data Availability

The data that support the findings of this study are available on request from the corresponding author. The data are not publicly available due to privacy or ethical restrictions.
