# Supplementary material for: Wrinkled Strain‐Enriched High‐Entropy Metallene Enables Cross‐Site Tandem Nitrate‐to‐Ammonia
Source: Angew Chem Int Ed Engl. 2026 May 12;65(27):e5828704. doi: 10.1002/anie.5828704 (PMC13327625; doi:10.1002/anie.5828704)
Supplement: Supplementary file 1 — Supporting File: anie72585‐sup‐0001‐SuppMat.docx. [file ANIE-65-e5828704-s001.docx]

**Supporting Information**

**Wrinkled Strain-Enriched High-Entropy Metallene Enables Cross-Site Tandem** **Nitrate-to-Ammonia**

Tianfang Yang^[a]^, Yang Liu*^[b]^, Menghao Kong^[b]^, Shilong Li^[a]^, Shizhe Liu^[a]^, Guanjie He*^[c]^, Shuyan Gao*^[a,b]^

[a] T. Yang, S. Li, S. Liu, Prof. S. Gao

School of Chemistry and Chemical Engineering, Henan Normal University, Xinxiang, Henan 453007, P. R. China

E-mail: shuyangao@htu.edu.cn

[b] Dr. Y. Liu, Prof. S. Gao

School of Materials Science and Engineering, Henan Normal University, Xinxiang, Henan 453007, PR China

E-mail: liuyang20208@htu.edu.cn

[c] Prof. G. He

Christopher Ingold Laboratory, Department of Chemistry, University College London, London, WC1H 0AJ, UK.

E-mail: g.he@ucl.ac.uk

1. **Experimental section**
   1. *Materials*

Palladium(II) acetylacetonate (Pd(acac)_2_), cobalt(Ⅲ) acetylacetonate (Co(acac)_2_, 98%), nickel(II) acetylacetonate (Ni(acac)_2_, 95%), copper(Ⅱ) acetylacetonate (Cu(acac)_2_,97%), iron acetylacetonate (Fe(acac)_2_, 98%), indium chloride (InCl_3_, 99.99%), N,N-dimethylformamide (DMF, 99.5%), acetic acid (99.8%), ascorbic acid (AA, 99%), tungsten hexacarbonyl (W(CO)_6_, 99.9%), molybdenum hexacarbonyl (Mo(CO)_6_, 98%), potassium nitrate (KNO_3_, 99%), potassium nitrate-15N (K^15^NO_3_, 99 at%), ammonium chloride (NH_4_Cl, 99.5%), ammonium chloride-15N (^15^NH_4_Cl, 99 at%), potassium sulfate (K_2_SO_4_, 99%), sodium hydroxide (NaOH, 96%), sodium hypochlorite (NaClO, ≥5.0% active chlorine basis), sodium nitroferricyanide(III) dihydrate (C_5_FeN_6_Na_2_O·2H_2_O), dimethyl sulfoxide-d6 (C_2_D_6_OS, 0.03% TMS), sulfanilamide (C_6_H_8_N_2_O_2_S, ≥99%), N-(1-naphthyl) ethylenediamine dihydrochloride (C_12_H_14_N_2_·2HCl, 98%), sulfamic acid (H_3_NO_3_S, ≥99.5%), tert-Butanol (C_4_H_10_O, ≥99.0%), sodium salicylate (C_7_H_5_NaO_3_, ≥99.5%) and maleic acid (C_4_H_4_O_4_, ≥99.0%) are all purchased from Aladdin Chemical Reagents Co., Ltd. All the chemicals are used directly without any further purification. The aqueous solutions in this work are prepared by using deionized water (18.25 MΩ.cm).

- 1. *Preparation of the catalysts*

**Synthesis of** **PdFeCoNiCuIn-HEM:** PdFeCoNiCuIn-HEM was fabricated through a wet chemical process. First, 8 mg of Pd(acac)_2_, 10 mg of Fe(acac)_2_, 10 mg of Co(acac)_2_, 10 mg of Ni(acac)_2_, 10 mg of Cu(acac)_2_, 10 mg of InCl_3_, 50 mg of ascorbic acid and 100 mg of W(CO)_6_ were dissolved in 8 mL of DMF and 2 mL of acetic acid, followed by stirring at room temperature for 6 h. After all solids are dissolved, the mixed solution is transferred into a 25 mL Teflon-lined stainless-steel autoclave, heated up to 160 ℃ and kept for 10 h. After the reaction solution naturally cooling down to room temperature, the product was washed with deionized water and ethanol several times, and dried at 80°C for future use.

**Synthesis of** **FeCoNiCu-MEA and** **PdFeCoNiCu-HEM**: The preparations of quinary PdFeCoNiCu-HEM and quaternary FeCoNiCu-MEA were similar to that of senary PdFeCoNiCuIn-HEM, except that the species of metal salt was different in the precursor solutions.

- 1. *Physic**al characterization*

Transmission electron microscopy (TEM), including high-resolution TEM (HRTEM) and high-angle annular dark-field (HAADF), along with corresponding energy-dispersive X-ray spectroscopy (EDS) mapping were performed using a JEOL JEM-F200 field emission transmission electron microscope. Atomic force microscopy (AFM) was employed on Bruker-Dimension Icon (Germany). X-ray diffraction (XRD) analysis was performed using a Bruker-D8 Focus diffractometer equipped with a Cu Kα radiation source (operated at 40 kV and 40 mA). X-ray photoelectron spectroscopy (XPS) measurements were conducted using an ESCALAB250Xi spectrometer (Thermo Fisher Scientific) featuring a monochromatic Al Kα X-ray source. X-ray Absorption Fine Structure (XAFS) was collected in fluorescence excitation mode at BL14W Beam line at the Shanghai Synchrotron Radiation Facility (SSRF) (Shanghai, China). The extended X-ray absorption fine structure (EXAFS) spectra were recorded in transmission mode. The XAFS spectra of standard samples were recorded in transmission mode and the energy was calibrated using metal foil standard. The spectra were processed and analyzed by the software codes Athena.^[1]^ The absorbance data was measured on an ultraviolet-visible (UV-Vis, TU-1810) spectrophotometer. ^1^H NMR spectra were collected on an AVANCE (III HD) 600 MHz nuclear magnetic resonance instrument (Bruker) using DMSO-d6 as the solvent, for isotope tracing experiments and determination of NH_3_ concentration. Electron paramagnetic resonance (EPR, EMX plus Bruker, Germany) analysis was used to verify the presence of active hydrogen (*H) using 5,5-dimethyl-1-pyrroline-N-oxide (DMPO) as the trapping agent. The in-situ attenuated total reflection Fourier-transform infrared spectroscopy (ATR-FTIR) was performed on a FT-IR spectrometer (Bruker VERTEX 70V) equipped with an MCT detector with silicon as the prismatic window. Differential electrochemical mass spectrometry (DEMS, HPR-40 DEMS) was employed for online measurements to capture and analyze reaction intermediates during NRA process.

- 1. *Electrochemical measurements*

All electrochemical experiments were performed using a three-electrode H-type cell separated by a Celgard 3501 ion-exchange membrane, connected to a CHI 660E electrochemical workstation (Chenhua, Shanghai). The cathode and anode compartments each contained 30 mL of Ar-purged electrolyte (0.1 M KNO_3_ + 0.5 M K_2_SO_4_). The catalyst-modified carbon paper (1 × 0.5 cm^2^, geometric area), saturated calomel electrode (SCE, KCl-saturated) and Pt foil (1 cm^2^) were used as the working electrode, reference electrode and counter electrode, respectively. The catalyst ink was prepared by dispersing 1.0 mg catalyst in the solution consisting of 10 µL 5 wt% Nafion solution and 190 µL deionized water and ethanol after ultrasonic treatment. All potentials were converted to the reversible hydrogen electrode (RHE) scale using: E (vs. RHE) = E (vs. SCE) + 0.241 V + 0.059pH. Linear sweep voltammetry (LSV) investigations were performed at a rate of 5 mV s^–1^ in 0.5 M aqueous K_2_SO_4_ solution with or without KNO_3_. Electrochemical Impedance Spectroscopy (EIS) was performed to study kinetics and mass transfer in the frequency range from 0.1 to 10^5^ Hz with a 5 mV amplitude at −0.15 V (vs. RHE). To measure electrochemical active surface area (ECSA) of the electrodes, the response current values and double-layer capacitance (C_dl_) were determined from the CV test with the scan rate of 30-180 mV s^−1^. The Chronoamperometry (CA) tests were performed three times at different potentials for 30min to obtain NH_3_ yield rate and Faraday efficiency (FE). Error bars represent standard deviations of NH_3_ yield and FE calculated from three independent measurements.

**Assembly of Zn-NO_3_^−^ battery and the electrochemical measurements.** The PdFeCoNiCuIn-HEM (1×1 cm^2^, mass loading: 1.0 mg cm**^−^**^2^) and Zn plate (2×2 cm^2^) were employed as the cathode and anode for Zn-NO_3_^−^ battery, respectively. A typical H-type cell with 30 mL of cathode electrolyte (0.1 M KNO_3_ + 0.5 M K_2_SO_4_) and 30 mL of anode electrolyte (6.0 M KOH + 0.2 M Zn(Ac)_2_) separated by a Nafion 117 ion-exchange membrane was assembled. The open circuit voltage (OCV) of the battery is determined by measuring the voltage change over time. For the discharging polarization curves, the scan rate of 5 mV s^−1^ is employed in the selected potential region. The galvanostatic discharge tests are conducted with various current densities (1, 2, 5, 10, and 20 mA cm^−2^). The cycling test is carried out with 40 min per discharge-charge cycle (20 min for charging and 20 min for discharging) with a scanning rate of 2 mV s^−1^. In addition, the power density (*P*) and specific capacity (*C*) are calculated according to the equations below:

$\text{P=IV}$ (eq1)

$\text{C=}\frac{\text{I×t}}{\text{m}}$ (eq2)

where *I* is the discharge current density and *V* is the corresponding voltage, *t* (h) is the service hours, *m* is the weight of the consumed zinc plate, the specific capacity is calculated from the galvanostatic discharge curve, normalized to the mass of consumed Zn anode.

- 1. *Determination of the concentration of N-containing species*

**Determination of** **NH_3_-N by UV-Vis.** The indophenol blue method on a UV-Vis spectrophotometer was employed for quantification of NH_3_-N.^[2]^ Chromogenic agent A was obtained by adding NaOH (0.5 g) to 100 mL of deionized water containing 1 mL of NaClO. Chromogenic agent B was prepared by adding C_7_H_5_NaO_3_ (5 g), NaOH (0.25 g), and C_5_FeN_6_Na_2_O·2H_2_O (0.02 g) into 50 mL of deionized water. A specific volume of electrolyte was sampled from the cathodic chamber and diluted to 10 mL, to which 0.5 mL of chromogenic agents A and B, respectively. After 60 min stabilization at room temperature, the UV-Vis absorbance of the mixed solution at a wavelength of 660 nm was recorded. The calibration curve for the determination of NH_3_-N was obtained using a series of standard NH_4_Cl solutions (**Figure S1**).

**Determination of NH_3_-N by NMR.** To unequivocally confirm that NH_3_ produced during NRA originates from nitrate reduction, isotope labeling experiments were performed using both ^14^KNO_3_ and ^15^KNO_3_ as nitrogen sources during chronoamperometric tests. Following electroreduction (at −0.55 V vs. RHE for 30 min), the electrolyte pH was adjusted to acidic conditions using 1 M H_2_SO_4_ to ensure the presence of detectable NH_4_^+^ ions. For quantitative ^1^H NMR analysis (600 MHz), a mixture was prepared by combining 900 µL of the acidified sample with 50 µL of C_2_D_6_OS (DMSO-d6) and an external standard (maleic acid, 0.0044 g for calibration standards). Calibration curves for ^14^NH_4_^+^/^15^NH_4_^+^ quantification were constructed using standard solutions of known concentrations, based on the peak area ratio of NH₄⁺ to maleic acid. The concentration of ^14^NH_4_^+^ or ^15^NH_4_^+^ in the electrocatalysis samples was then determined by comparing the measured NH_4_⁺ peak area ratio to the corresponding calibration curve (**Figure S2**).

Calculation of NH_3_ yield rate and Faradaic efficiency. The NH_3_ yield rate ($\text{Y}_{\text{N}\text{H}_{\text{3}}}$) and Faradaic efficiency were determined by the following equations:^[3]^

$\text{F}\text{E}_{\text{N}\text{H}_{\text{3}}}\text{ }\text{=}\text{ }\text{(8}\text{ }\text{×}\text{ }\text{F}\text{ }\text{×}\text{ }\text{C}_{\text{N}\text{H}_{\text{3}}}\text{ }\text{×}\text{ }\text{V)}\text{ }\text{/}\text{ }\text{Q}$ (eq3)

$\text{Y}_{\text{N}\text{H}_{\text{3}}}\text{ }\text{=}\text{ }\text{(}\text{C}_{\text{N}\text{H}_{\text{3}}}\text{ }\text{×}\text{ }\text{V)}\text{ }\text{/}\text{ }\text{(}\text{ }\text{t}\text{ }\text{×}\text{ }\text{S)}$ (eq4)

where F is Faradaic constant (96485 C mol^−1^), $\text{C}_{\text{N}\text{H}_{\text{3}}}$ represents the concentration of NH_3_, V is the volume of electrolyte in the cathode compartment (30 mL), Q is the total amount of charge (C), t is the electrolysis time (0.5 h), S is the geometric area of the working electrode (0.5 cm^2^).

- 1. **H detection using EPR experiments*

Electron paramagnetic resonance (EPR) spectroscopy was employed to detect and identify radical intermediates generated during electrocatalysis, utilizing 5,5-dimethyl-1-pyrroline-N-oxide (DMPO) as a spin-trapping agent. Experiments were conducted using a three-electrode electrochemical cell configuration integrated with the EPR system. The potentiostatic electrolysis was carried out in 0.5 M K_2_SO_4_ solution with or without 0.1 M KNO_3_ at −0.55 V vs. RHE for 5 min. Immediately following electrolysis, 1 mL of electrolyte was sampled from the cathode chamber and rapidly mixed with 10 μL of neat DMPO. This mixture was then transferred into quartz capillary tubes for EPR analysis. All EPR spectra were acquired using a Bruker EMXplus spectrometer operating in the X-band at a microwave frequency of 9.8 GHz with a sweep width of 100 G centered at a magnetic field of 3510 G.

- 1. *In* *situ ATR-FTIR*

Measurements utilized a Bruker VERTEX FTIR spectrometer equipped with a liquid nitrogen-cooled mercury-cadmium-telluride (MCT) detector. The silicon crystal, coated with a thin gold film, served as the reflecting element and working electrode substrate. A catalyst ink dispersion (in ethanol) was deposited onto this Au/Si surface. The electrochemical cell comprised this modified working electrode, an Ag/AgCl reference electrode, and a carbon rod counter electrode. In-situ ATR-FTIR was conducted in a 0.1 M KNO_3_ + 0.5 M K_2_SO_4_electrolyte solution, with the applied potential scanned from the open-circuit potential (OCP) to -0.80 V vs. RHE. Spectra across the 1000-4000 cm^−1^ range were collected every 30 seconds. All spectra were processed using background correction relative to a reference spectrum.

- 1. *DEMS measurements*

The mass spectrometer coupled with an electrochemical workstation was employed for in situ DEMS measurements. An electrochemical cell, designed for the experiment, was continuously supplied with an electrolyte solution (0.1 M KNO_3_ + 0.5 M K_2_SO_4_) via a peristaltic pump. An inert atmosphere was maintained throughout the experiment by constant bubbling of Ar gas into the electrolyte. The working electrode was prepared as a self-supported structure, with a Pt wire serving as the counter electrode and an Ag/AgCl electrode (saturated KCl) as the reference electrode. Linear Sweep Voltammetry (LSV) was performed from 0 to −0.9 V vs. RHE at a scan rate of 8 mV s^−1^. After each LSV test, the system was allowed to stabilize until the mass signal intensity returned to baseline, at which point a new cycle was initiated. A total of six cycles were conducted to ensure reliable data collection and minimize any potential experimental artifacts.

- 1. *Theoretical calculation*

First-principles density functional theory (DFT) calculations were performed using the Vienna Ab Initio Simulation Package (VASP) with projector augmented wave (PAW) pseudopotentials and the Perdew-Burke-Ernzerhof (PBE) generalized gradient approximation (GGA).^[4]^ A plane-wave basis set with a kinetic energy cutoff of 500 eV was employed. Structural optimizations required atomic forces to converge below 0.02 eV Å^−1^, and self-consistent field calculations utilized an energy convergence threshold of 10^‒5^ eV. The computational models employed four-layer 3×3 supercells separated by a 20 Å vacuum layer to mitigate periodic boundary effects, with dipole corrections applied to eliminate spurious electrostatic interactions. Dispersion forces were incorporated via the DFT-D3 method, while solvation effects critical for simulating aqueous-phase hydrogen transfer processes (*H) were modeled implicitly using VASPsol. This integrated approach systematically addresses electronic structure relaxation, long-range interactions, and electrochemical interfacial environments. The free energy of surface adsorbates is calculated according to the equation below:

$\text{∆G = ∆}\text{E }\text{+ ∆}\text{E}_{\text{ZPE}}\text{ }\mathbf{-}\text{ T}\text{∆}\text{S}$ (eq5)

where ΔE is the reaction energy obtained by the total energy difference between the reactant and product molecules absorbed on the catalyst surface, ΔE_ZPE_ is the zero-point energy, and $\text{∆S}$ is estimated through molecular vibration analysis under harmonic approximation, T is the Fahrenheit temperature (currently 298.15K).

1. **Figure captions**


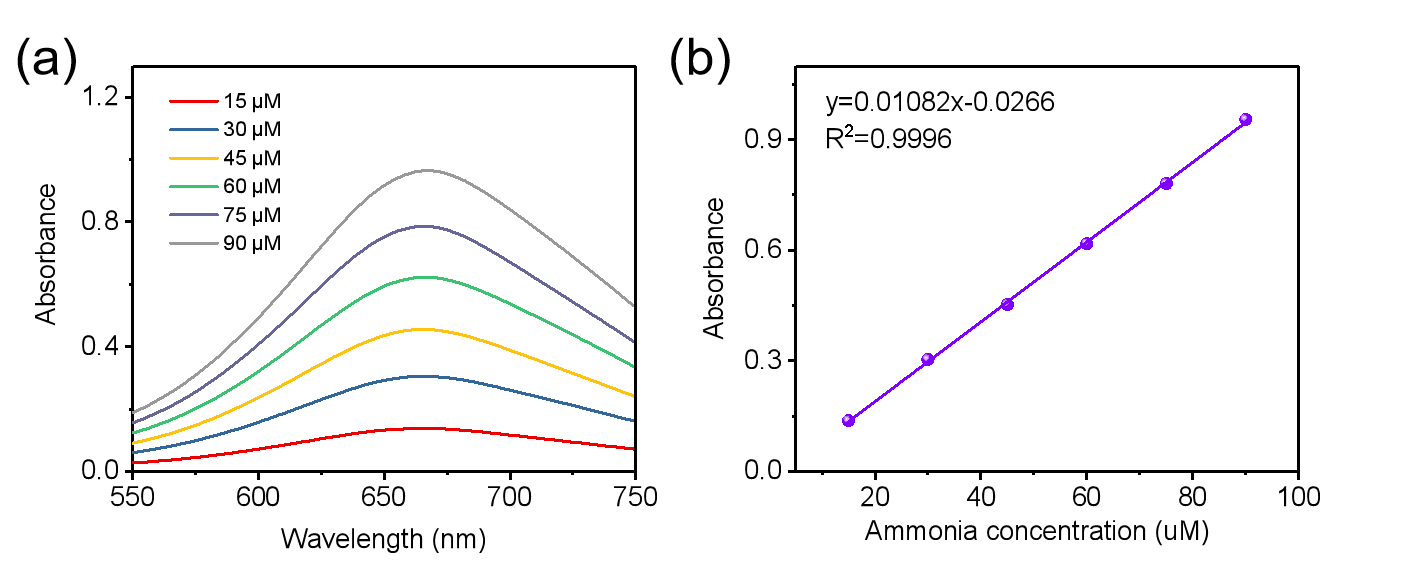


**Figure S1.** (a) The UV-Vis absorption spectra of NH_4_Cl standard solution with varying concentrations, (b) absorbance-NH_4_^+^ concentration fitting.


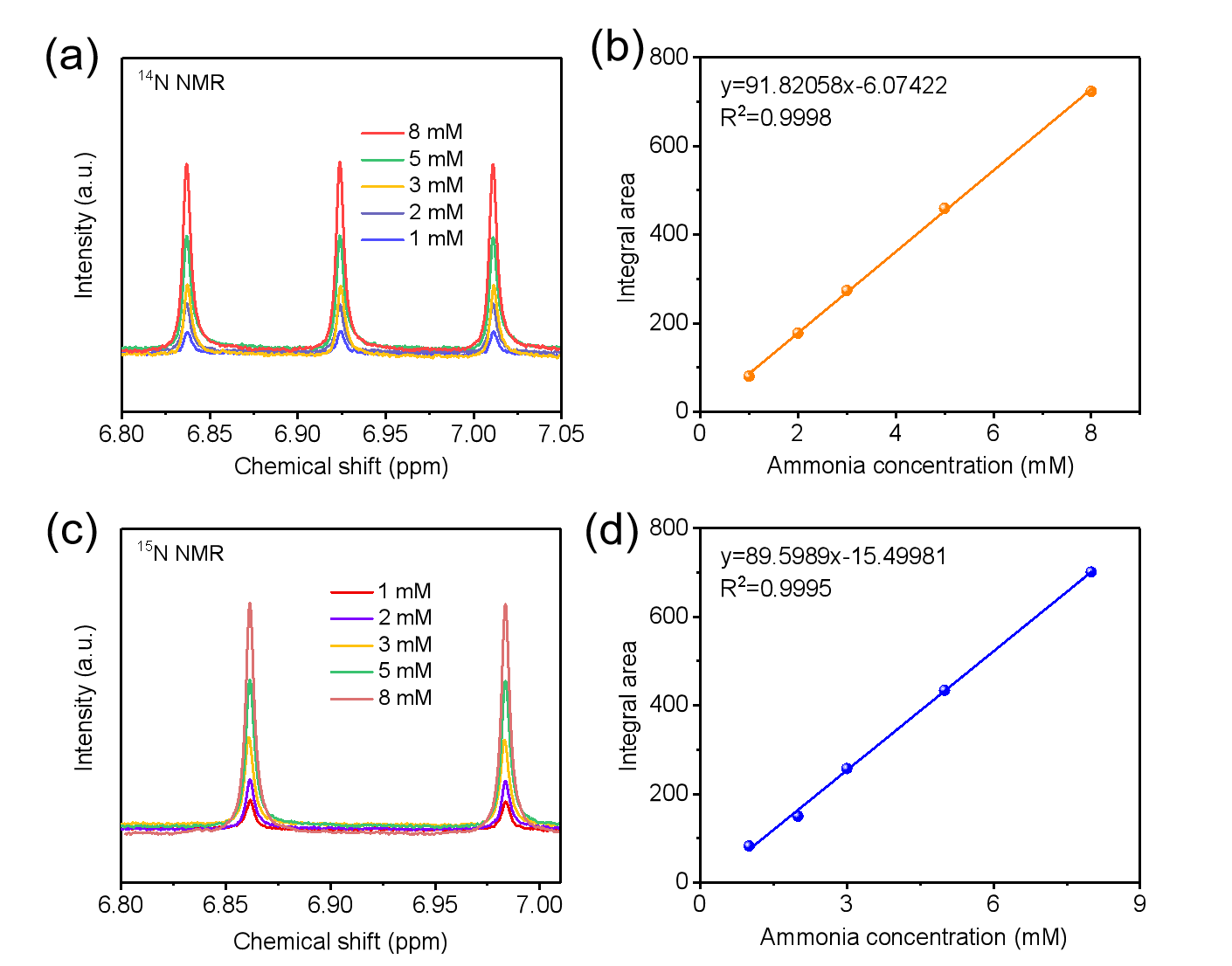


**Figure S2.** (a) ^1^H NMR spectra (600 MHz) of a series of standard ^14^NH_4_^+^ solutions with different concentrations. (b) The standard curve of integral area (^14^NH_4_^+^-14N/C_4_H_4_O_4_) against ^14^NH_4_^+^-^14^N concentration. (c) ^1^H NMR spectra (600 MHz) of a series of standard ^15^NH_4_^+^ solutions with different concentrations. (d) The standard curve of integral area (^15^NH_4_^+^-^15^N/C_4_H_4_O_4_) against ^15^NH_4_^+^-^15^N concentration.

**
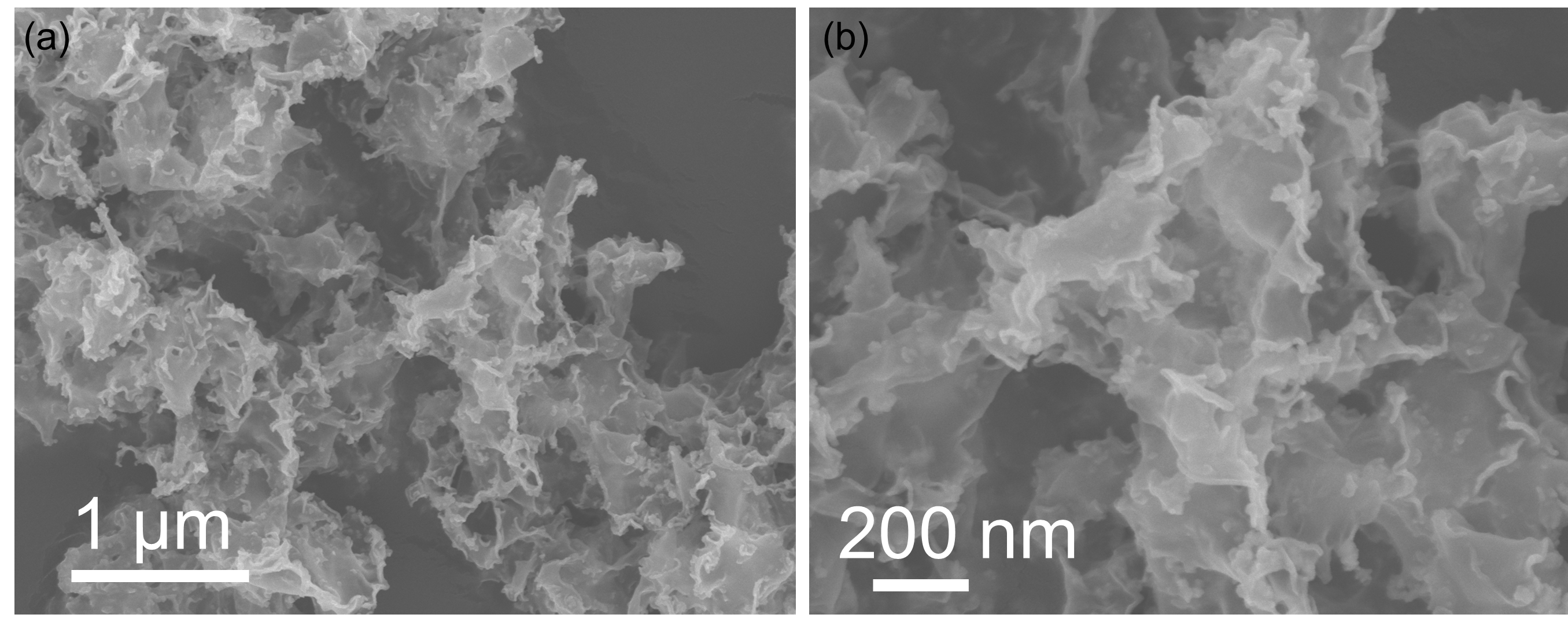
**

**Figure S3.** SEM images of PdFeCoNiCuIn-HEM.

**Figure S4.** XRD spectra of PdFeCoNiCu and PdFeCoNiCuIn-HEM, orange dashed lines: PDF#46-1043.


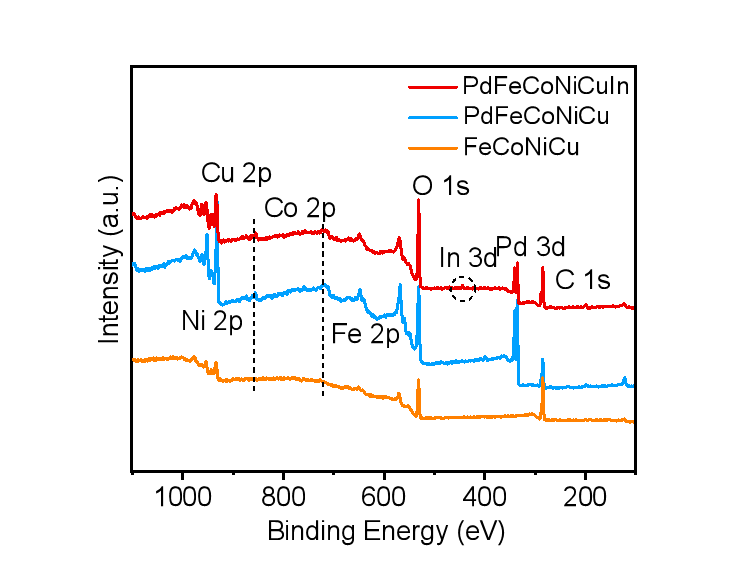


**Figure S5.** XPS survey spectra of FeCoNiCu-MEA, PdFeCoNiCu, and PdFeCoNiCuIn-HEM.


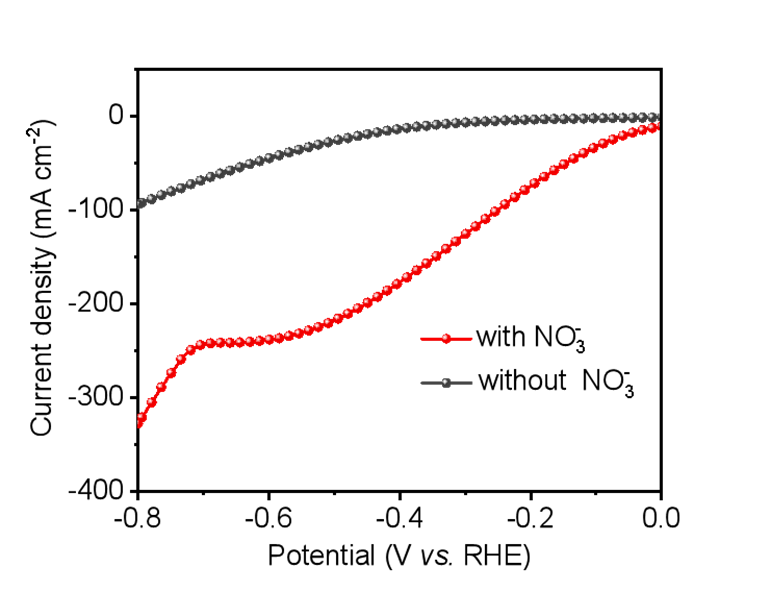


**Figure S6.** LSV curves of PdFeCoNiCuIn-HEM 0.5 M K_2_SO_4_ with and without 0.1 M KNO_3_ solution.


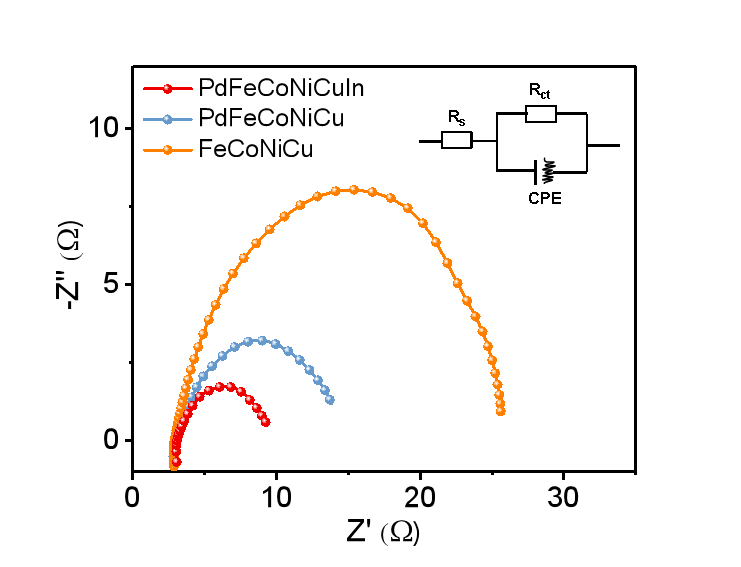


**Figure S7.** EIS spectra of FeCoNiCu-MEA, PdFeCoNiCu-HEM and PdFeCoNiCuIn-HEM.


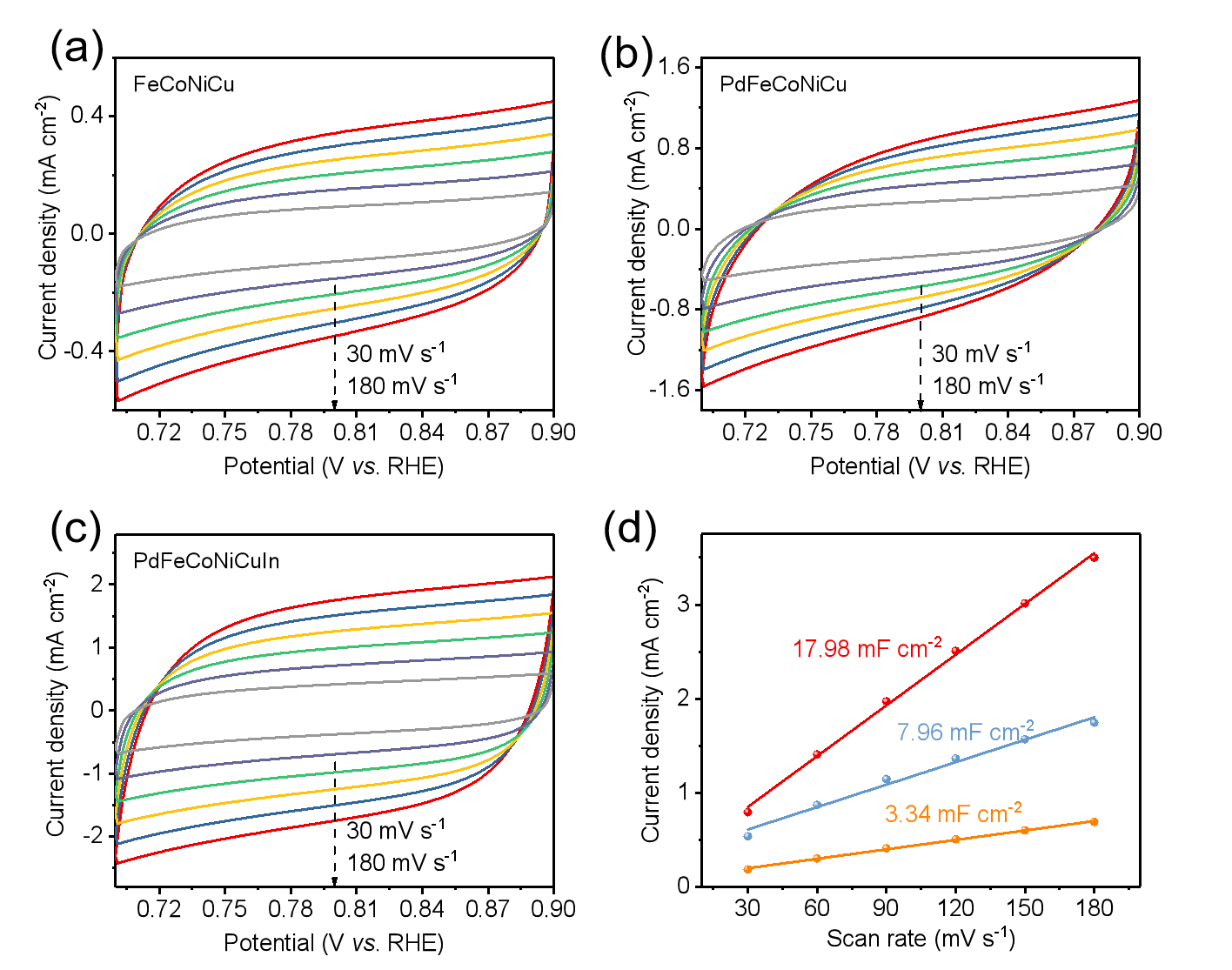


**Figure S8.** The measurement of electrochemical double-layer capacitance (C_dl_). CV curves of (a) FeCoNiCu-MEA, (b) PdFeCoNiCu-HEM, (c) PdFeCoNiCuIn-HEM, (d) C_dl_ values.


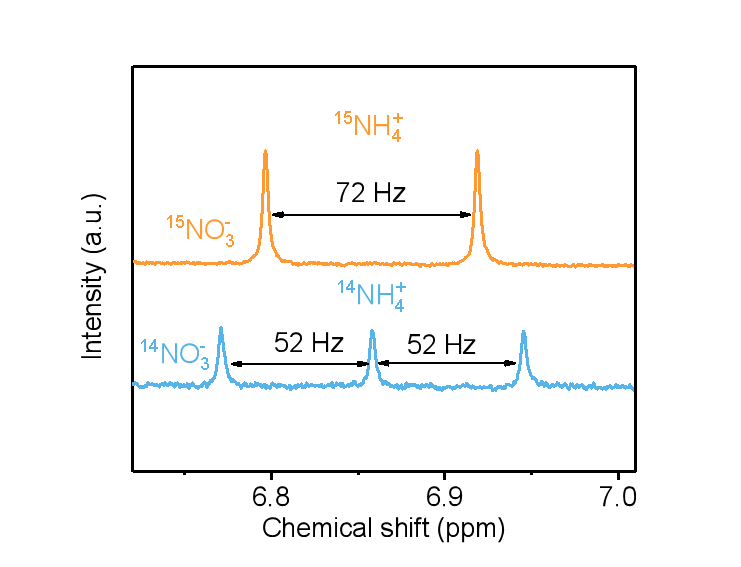


**Figure S9.** ^1^H NMR spectra of the electrolyte performed by PdFeCoNiCuIn-HEM using ^14^NO_3_^–^ and ^15^NO_3_^–^ as N-source.


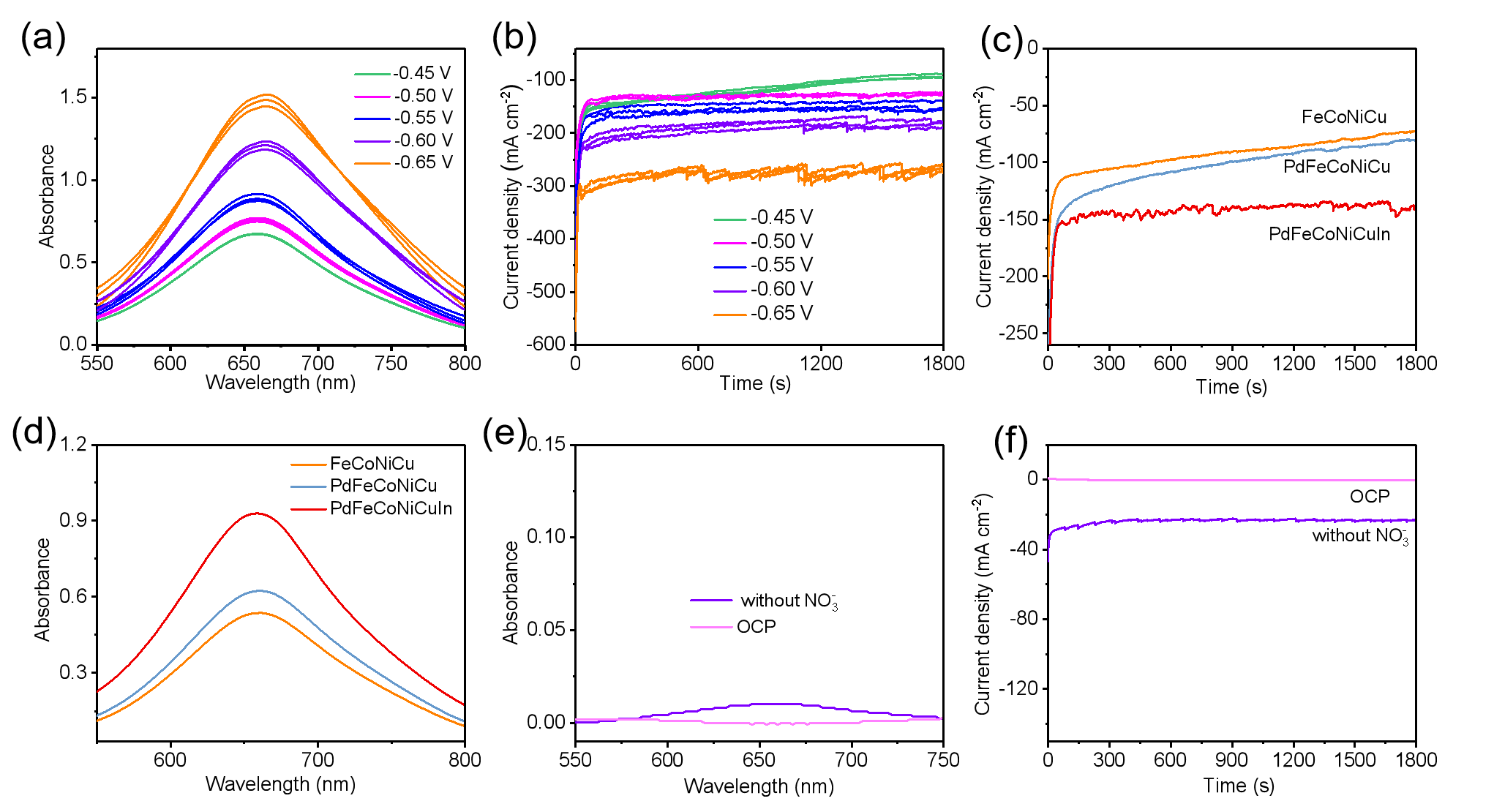


**Figure S10.** (a) CA curves after 30 min NRA in 0.5 M K_2_SO_4_ + 0.1 M KNO_3_ at different potentials, (b) UV-Vis absorption spectra of PdFeCoNiCuIn-HEMs. (c, d) CA curves and UV-Vis absorption spectra of PdFeCoNiCu and FeCoNiCu. (e) CA curves, (f) UV-Vis absorption spectra of PdFeCoNiCuIn-HEMs in 0.5 M K_2_SO_4_ without NO_3_^−^ and OCP.


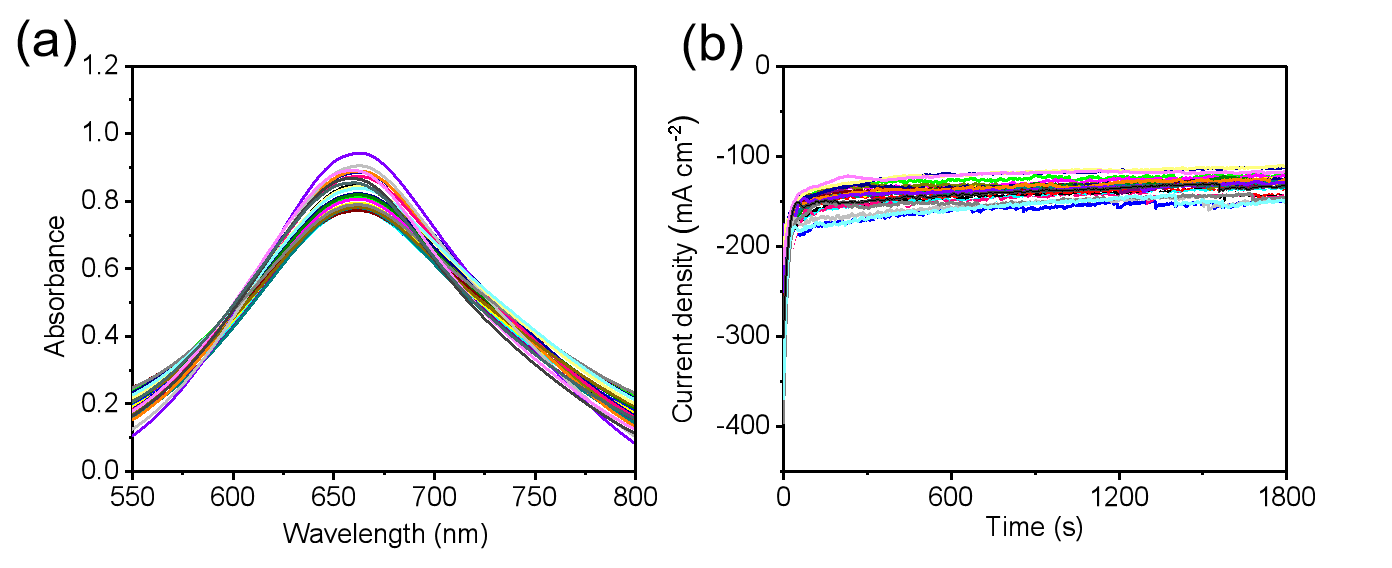


**Figure S11.** Cyclic stability tests of PdFeCoNiCuIn-HEMs: (a) CA curves, (b) UV-Vis absorption spectra.


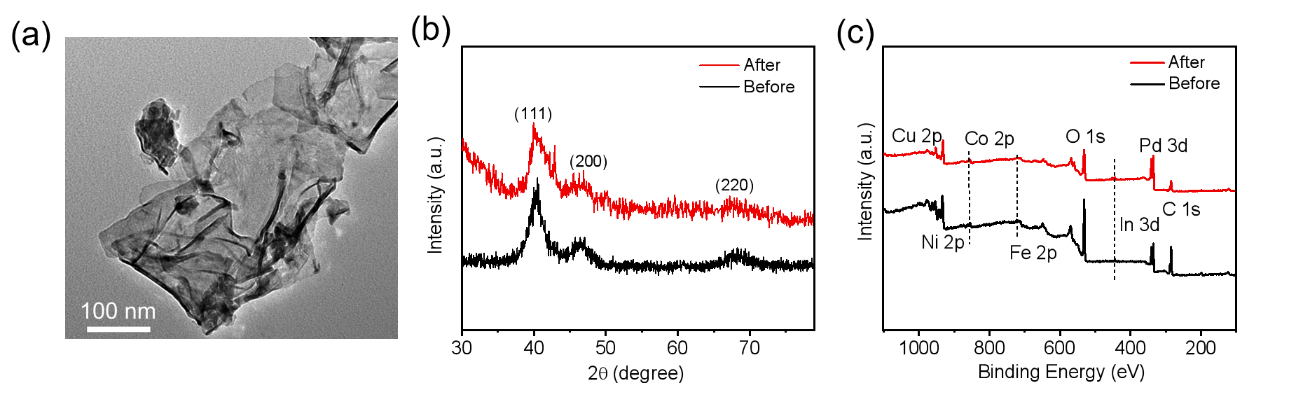


**Figure S12.** (a) TEM image, (b) XRD patterns, (c) XPS survey spectra of PdFeCoNiCuIn-HEM after electrochemical testing.


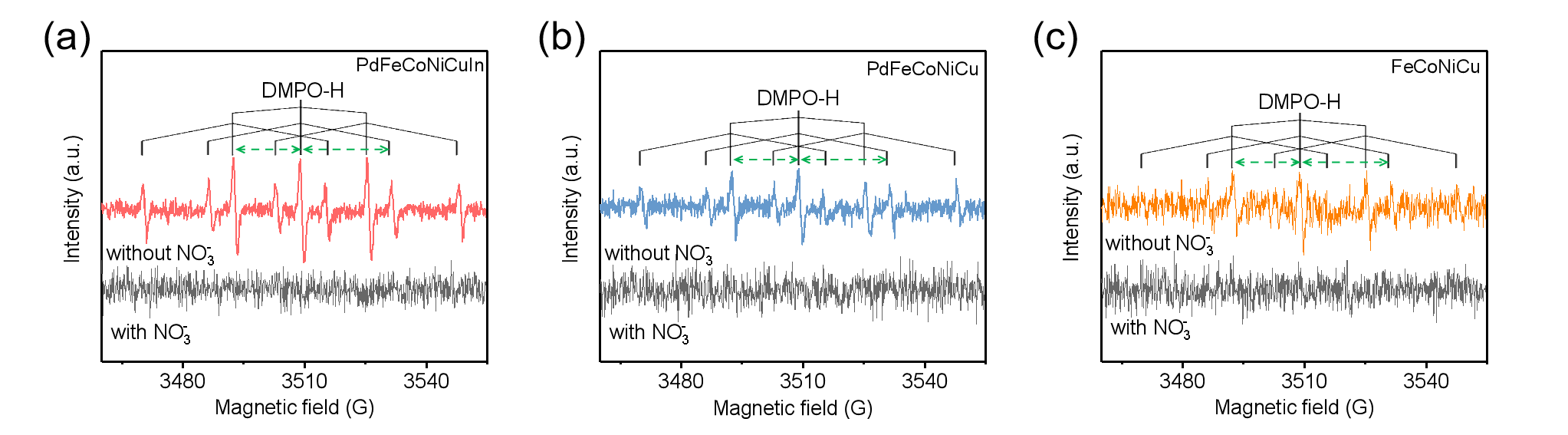


**Figure S13.** (a-c) DMPO-involved EPR spectra of PdFeCoNiCuIn-HEM, PdFeCoNiCu-HEM and FeCoNiCu-MEA under different electrolysis conditions.


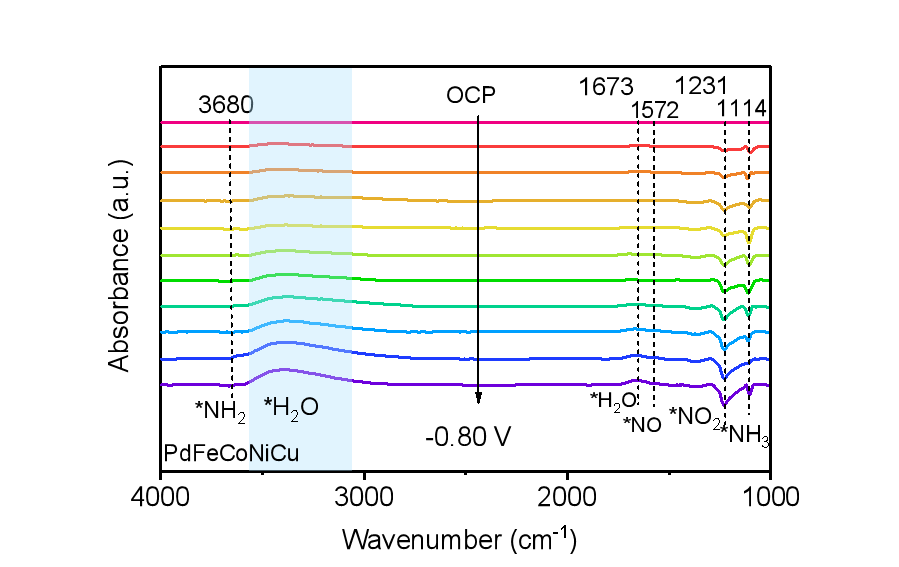


**Figure S14**. In situ ATR-FTIR spectra at different potential PdFeCoNiCu-HEM.


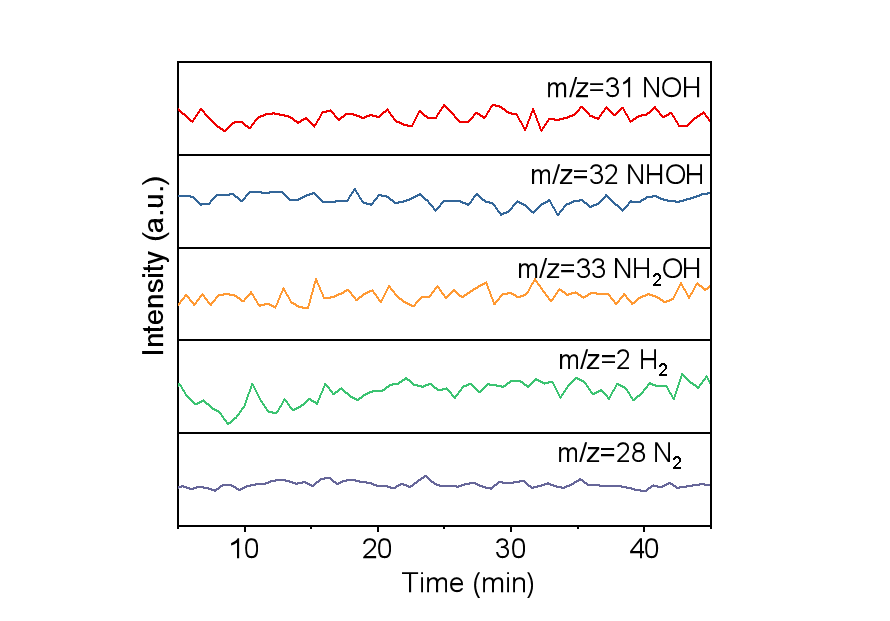


**Figure S15.** Online DEMS measurements of PdFeCoNiCuIn-HEM, signals at m/z = 33 (NH_2_OH), 32 (NHOH), 31 (NOH), 28 (N_2_), 2 (H_2_).


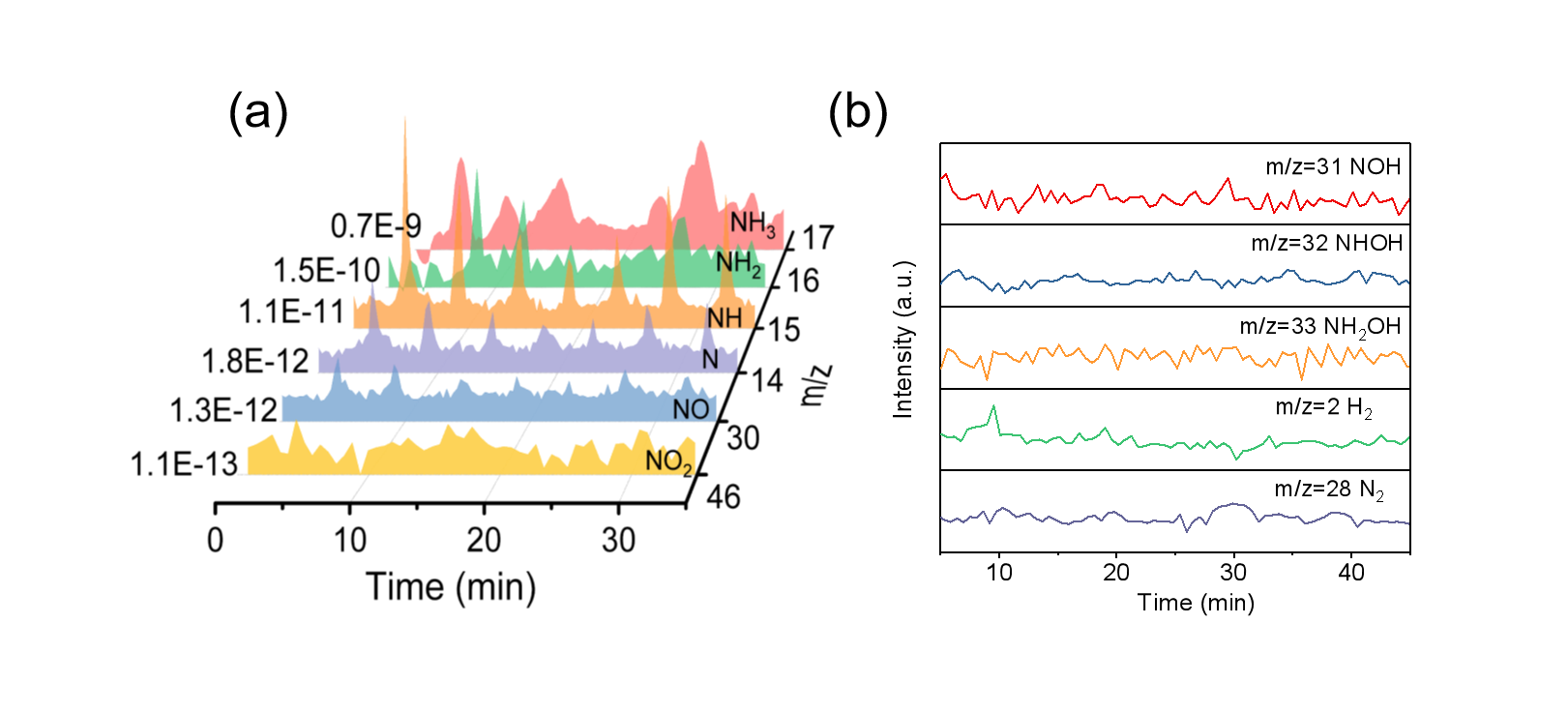


**Figure S16.** Online DEMS measurements of PdFeCoNiCu-HEM.


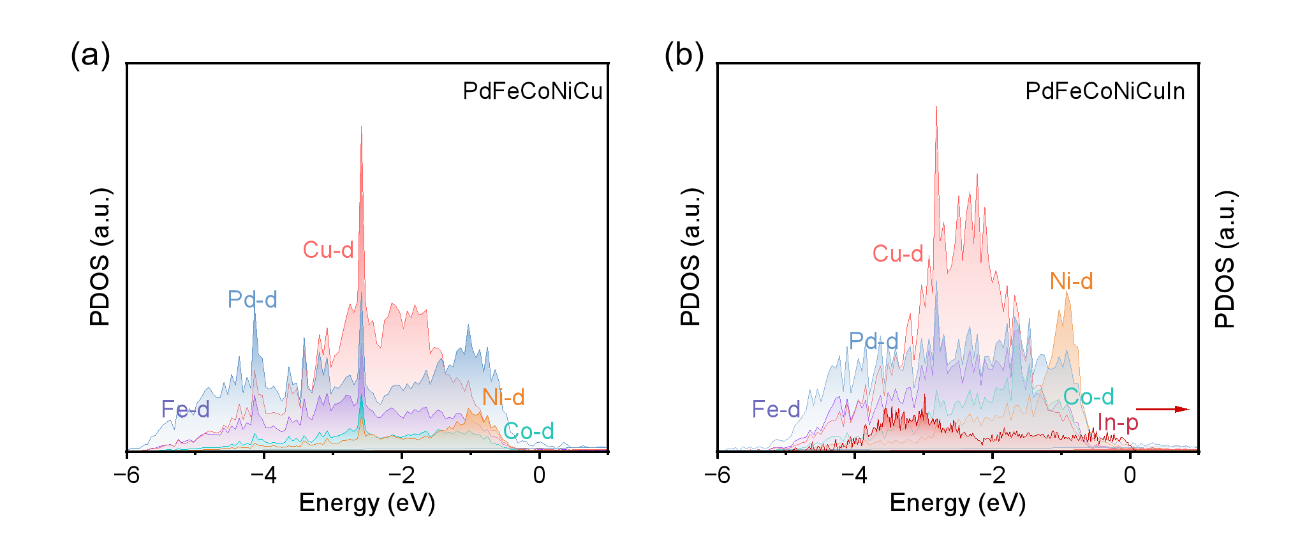


**Figure S17.** Projected density of states (PDOSs) of all the elements in the PdFeCoNiCu-HEM and PdFeCoNiCuIn-HEM.


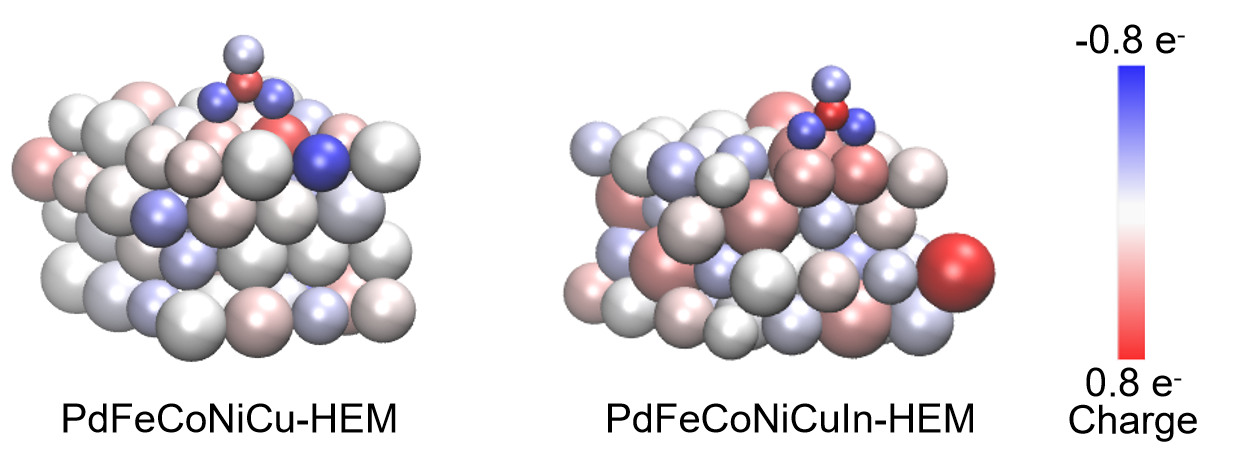


**Figure S18.** Bader charge analysis of adsorbed NO_3_^−^ on PdFeCoNiCu-HEM and PdFeCoNiCuIn-HEM. The positive (negative) charge indicates electron gain (loss).

**
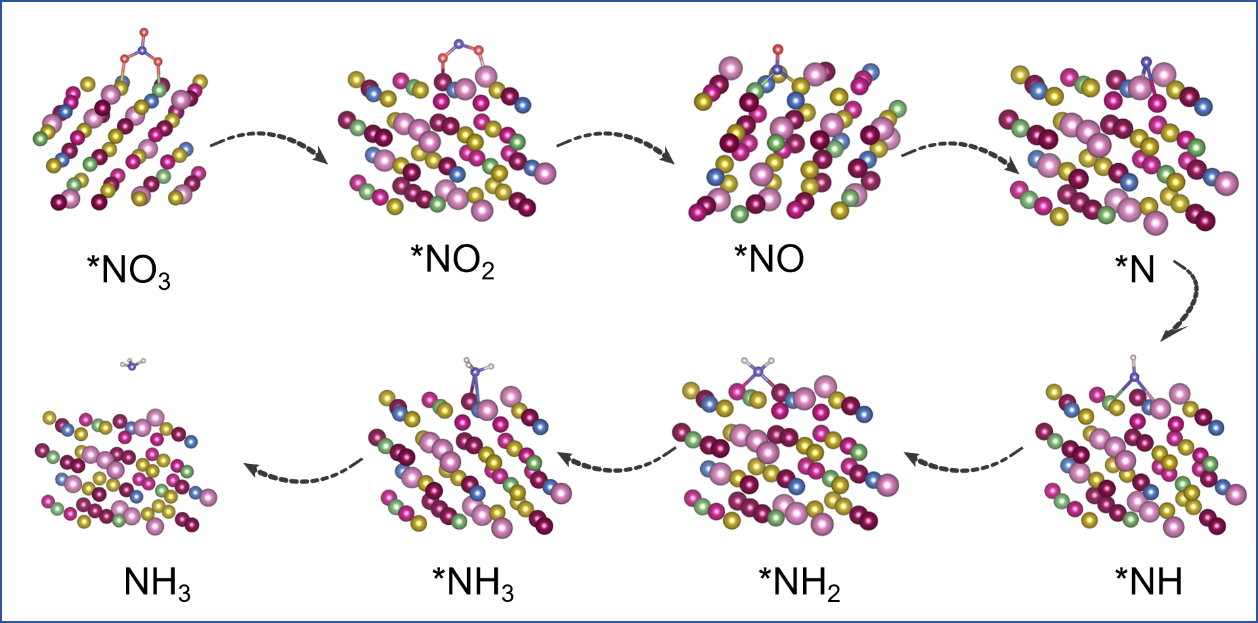
**

**Figure S19.** The adsorption models of various intermediates along the optimal tandem catalytic pathway (pathway 2) during NRA.

**Figure S20.** Power density curves at different catalyst mass loadings.


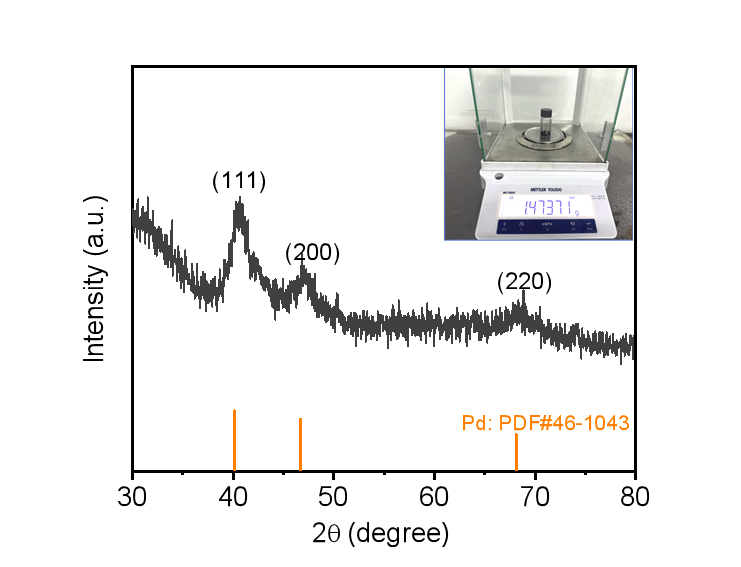


**Figure S21.** XRD pattern of the mass-produced PdFeCoNiCuIn-HEM catalyst at the gram-scale.


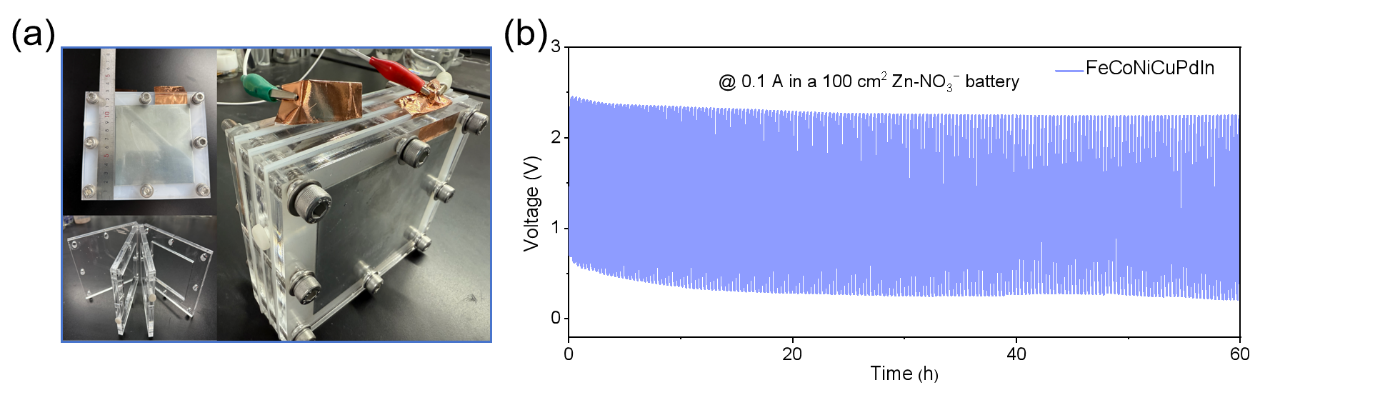


**Figure S22.** (a) Digital photographs of the assembled large-area 10 × 10 cm^2^ Zn-NO_3_⁻ battery. (b) Galvanostatic discharge-charge cycling stability of the large-area battery operating at a high current of 0.1 A for 60 h.

**Table S1** The element contents of PdFeCoNiCuIn-HEM.

| Element | Content (at%) | Content (Normalized at%) |
| --- | --- | --- |
| Fe | 3.54 | 16.14 |
| Co | 2.28 | 10.40 |
| Ni | 2.52 | 11.49 |
| Cu | 6.08 | 27.72 |
| Pd | 2.46 | 11.22 |
| In | 5.05 | 23.03 |

**Table S2**. EIS fitting parameters of FeCoNiCu, PdFeCoNiCu and PdFeCoNiCuIn-HEM.

| **Catalysts** | ***R*_ct_ (Ω)** | ***R*_s_ (Ω)** |
| --- | --- | --- |
| FeCoNiCu | 23.40 | 3.27 |
| PdFeCoNiCu | 11.81 | 3.64 |
| PdFeCoNiCuIn | 6.54 | 3.56 |

**Table S3.** The NRA performances of FeCoNiCu, PdFeCoNiCu and PdFeCoNiCuIn-HEM.

| **Catalyst** | **NH_3_ yield** | **Faradaic efficiency (%)** |
| --- | --- | --- |
| FeCoNiCu | 383.0 μmol⋅h^–1^⋅cm^–2^ | 88.5 |
| PdFeCoNiCu | 443.0 μmol⋅h^–1^⋅cm^–2^ | 91.0 |
| PdFeCoNiCuIn | 665.3 μmol⋅h^–1^⋅cm^–2^ | 99.3 |

**Table S4**. The comparison of NRA performances.

| **Catalysts** | **Electrolyte** | **Potential vs. RHE (V)** | **NH_3_ FE**  **(%)** | **NH_3_ yield rate**  **(μmol h^–1^ cm^–2^)** | **Ref.** |
| --- | --- | --- | --- | --- | --- |
| PdFeCoNiCuIn-HEM | 0.5 M K_2_SO_4_  0.1 M KNO_3_ | -0.55 | 99.3 | 665.3 | This work |
| CoPhz/CNT | 0.1 M Na_2_SO_4_  0.1 M KNO_3_ | -1.15 | 93 | 491.1 | [5] |
| CuO@PANI/CF | 0.5 M K_2_SO_4_  200 ppm NO_3_^−^-N | -0.65 | 93.88 | 213 | [6] |
| Ru-Fe_2_O_3_ | 0.5 M Na_2_SO_4_  0.1 M NO_3_^−^ | -0.9 | 72.8 | 329.4 | [7] |
| Ni_1_Cu-SAA | 0.5 M K_2_SO_4_  200 ppm NO_3_^−^-N | -1.0 | 95 | 1176.5 | [8] |
| Cd_SA_-Mo_2_TiC_2_Tx | 0.1 M KNO_3_  0.2 M Na_2_SO_4_ | -0.5 | 95.00 | 285 | [9] |
| Co_4_Fe_6_ | 0.1M Na_2_SO_4_  200 ppm NO_3_^−^-N | -0.69 | 98.6 | 159.3 | [10] |
| Cu_2_O@Py‑COF | 0.1 M Na_2_SO_4_  0.1 M KNO_3_ | -0.7 | 84 | 135.3 | [11] |
| 1D MnFeO_x_ | 0.5 M K_2_SO_4_  0.05 M KNO_3_ | -1.0 | 95.9 | 723.5 | [12] |
| Cu-SNAs | 0.5 M PBS  0.1 M NaNO_3_ | -0.55 | 88 | ~250 | [13] |
| Ni_0.25_Cu_0.5_Sn_0.25_ | 0.5 M Na_2_SO_4_  0.1 M KNO_3_ | -0.2 | 98.42 | 213.86 | [14] |
| Cu_10_Fe_1_-CFP | 0.5 M Na_2_SO_4_  0.1 M KNO_3_ | -0.35 | 93.74 | 190.45 | [15] |
| Bi-CuCo_2_O_4_ | 0.5 M Na_2_SO_4_  0.1 M KNO_3_ | -0.8 | 95.53 | 448.74 | [16] |

**Table S5.** The free energy of each step of nitrate reduction of different active sites in quinary PdFeCoNiCu-HEM.

|  | CoCo | CoCu | CoNi | CoPd | CuCu | CuPd | FeCo | FeCu | FePd | NiCu | NiNi | NiPd |
| --- | --- | --- | --- | --- | --- | --- | --- | --- | --- | --- | --- | --- |
| NO_3_^–^ | 0.00 | 0.00 | 0.00 | 0.00 | 0.00 | 0.00 | 0.00 | 0.00 | 0.00 | 0.00 | 0.00 | 0.00 |
| NO_3_^−^→*NO_3_ | -0.37 | -0.11 | -0.27 | -0.01 | -0.18 | 0.22 | -0.43 | -0.19 | -0.19 | -0.01 | -0.17 | 0.05 |
| *NO_3_→*NO_2_ | -1.59 | -0.21 | -1.83 | -1.86 | -1.46 | -0.97 | -1.82 | -1.76 | -1.83 | -1.87 | -1.70 | -1.89 |
| *NO_2_→*NO | -0.67 | -2.84 | -2.14 | -0.53 | -0.45 | -2.41 | -0.60 | -1.15 | 0.63 | -0.75 | -0.62 | -0.17 |
| *NO→*N | -2.96 | -1.84 | -1.07 | -1.79 | 0.35 | -0.29 | -2.11 | -0.42 | -3.57 | 0.76 | -2.81 | -2.13 |
| *N→*NH | -0.34 | -0.24 | 0.94 | -0.59 | -2.56 | 1.88 | -0.94 | -1.68 | 1.41 | -1.23 | -0.15 | 1.16 |
| *NH→*NH_2_ | 0.05 | -0.47 | -1.50 | -1.09 | -1.41 | -4.01 | -0.02 | -0.39 | -2.11 | -1.47 | -0.39 | -2.55 |
| *NH_2_→*NH_3_ | -0.26 | -0.43 | -0.20 | 0.70 | -0.16 | -0.32 | -0.16 | -0.56 | -0.49 | -1.51 | -0.27 | 0.23 |
| *NH_3_→NH_3_ | 0.73 | 0.73 | 0.67 | -0.24 | 0.45 | 0.49 | 0.67 | 0.74 | 0.73 | 0.66 | 0.70 | -0.10 |

**Table S6.** The free energy of each step of nitrate reduction of different active sites in PdFeCoNiCuIn-HEM.

|  | | CoCu | | CoIn | | CoNi | | CoPd | | CuCu | | CuIn | | CuPd | FeCo | |
| --- | --- | --- | --- | --- | --- | --- | --- | --- | --- | --- | --- | --- | --- | --- | --- | --- |
| NO_3_^–^ | | 0.00 | | 0.00 | | 0.00 | | 0.00 | | 0.00 | | 0.00 | | 0.00 | 0.00 | |
| NO_3_^−^→*NO_3_ | | -0.49 | | -0.66 | | -0.86 | | 0.10 | | -1.03 | | -0.43 | | -0.17 | -0.82 | |
| *NO_3_→*NO_2_ | | -2.13 | | -1.75 | | -1.73 | | -2.69 | | -1.44 | | -1.55 | | -1.79 | -1.72 | |
| *NO_2_→*NO | | -1.75 | | -1.96 | | -1.88 | | -1.88 | | -2.03 | | -2.51 | | -2.50 | -1.75 | |
| *NO→*N | | -0.20 | | -0.20 | | -0.96 | | -0.79 | | -0.74 | | -0.08 | | 1.08 | -0.99 | |
| *N→*NH | | -0.72 | | -1.36 | | -0.50 | | -0.39 | | -0.76 | | -0.72 | | -2.96 | -0.52 | |
| *NH→*NH_2_ | | -0.22 | | 0.25 | | -0.11 | | 0.13 | | -0.12 | | 0.04 | | 1.10 | 0.18 | |
| *NH_2_→*NH_3_ | | -0.61 | | 0.61 | | -0.09 | | -0.47 | | 0.09 | | -0.43 | | -0.54 | -0.49 | |
| *NH_3_→NH_3_ | | 0.72 | | -0.34 | | 0.73 | | 0.58 | | 0.60 | | 0.27 | | 0.36 | 0.71 | |
|  | FeCu | | FeFe | | FeIn | | FeNi | | FePd | | NiCu | | NiIn | NiPd | PdIn |  |
| NO_3_^–^ | 0.00 | | 0.00 | | 0.00 | | 0.00 | | 0.00 | | 0.00 | | 0.00 | 0.00 | 0.00 |  |
| NO_3_^−^→*NO_3_ | -0.86 | | -1.04 | | -0.36 | | -0.99 | | -0.64 | | -0.79 | | -0.58 | -0.91 | 0.03 |  |
| *NO_3_→*NO_2_ | -1.77 | | -1.74 | | -1.75 | | -1.71 | | -1.52 | | -1.78 | | -2.10 | -1.44 | -0.82 |  |
| *NO_2_→*NO | -1.91 | | -1.90 | | -2.56 | | -1.75 | | -1.94 | | -1.31 | | -1.77 | -2.25 | -3.50 |  |
| *NO→*N | -1.15 | | -1.21 | | -0.53 | | -0.94 | | -1.14 | | -1.56 | | -0.98 | -0.94 | -0.97 |  |
| *N→*NH | -0.63 | | -0.46 | | -0.59 | | -0.51 | | -0.37 | | -0.50 | | -0.45 | 0.66 | 0.33 | |
| *NH→*NH_2_ | 0.04 | | 0.00 | | -0.25 | | -0.43 | | -0.71 | | -0.14 | | 0.20 | -1.42 | -1.01 | |
| *NH_2_→*NH_3_ | 0.06 | | 0.04 | | -0.07 | | 1.24 | | 0.67 | | -0.08 | | -0.29 | 1.24 | 0.34 | |
| *NH_3_→NH_3_ | 0.80 | | 0.91 | | 0.69 | | -0.32 | | 0.24 | | 0.75 | | 0.55 | -0.35 | 0.19 | |

**Table S7.** Tandem catalytic pathways with different combinations of active sites (Ni-In → Pd-In → Ni-Cu → Cu-Cu → Fe-In → Fe-Ni → Co-Cu → Co-In, MSE = 0.078).

|  | Active Site | ΔG (eV) |
| --- | --- | --- |
| NO_3_^−^→*NO_3_ | Ni-In | -0.58 |
| *NO_3_→*NO_2_ | Pd-In | -0.82 |
| *NO_2_→*NO | Ni-Cu | -1.31 |
| *NO→*N | Cu-Cu | -0.74 |
| *N→*NH | Fe-In | -0.59 |
| *NH→*NH_2_ | Fe-Ni | -0.43 |
| *NH_2_→*NH_3_ | Co-Cu | -0.61 |
| *NH_3_→NH_3_ | Co-In | -0.34 |

**Table S8.** Tandem catalytic pathways with different combinations of active sites (Ni-Cu → Pd-In → Ni-Cu → Fe-In → Ni-In → Fe-Pd → Co-Pd → Co-In, MSE = 0.12).

|  | Active Site | ΔG (eV) |
| --- | --- | --- |
| NO_3_^−^→*NO_3_ | Ni-Cu | -0.79 |
| *NO_3_→*NO_2_ | Pd-In | -0.82 |
| *NO_2_→*NO | Ni-Cu | -1.31 |
| *NO→*N | Fe-In | -0.53 |
| *N→*NH | Ni-In | -0.45 |
| *NH→*NH_2_ | Fe-Pd | -0.71 |
| *NH_2_→*NH_3_ | Co-Pd | -0.47 |
| *NH_3_→NH_3_ | Co-In | -0.34 |

**Table S9**. Comparison of the Zn-NO_3_^−^ performance of PdFeCoNiCuIn-HEM with recently reported Zn-NO_3_^−^ batteries.

| **Catalysts** | **OCV (V)** | **Power density**  **(mW cm^−2^)** | **NH_3_ yield rate** | **Stability (h)** | **Ref.** |
| --- | --- | --- | --- | --- | --- |
| PdFeCoNiCuIn-HEM | 1.48 | 7.36 | 112.8 μmol h^−1^ cm^−2^ | 100 | This work |
| Ni-MOF-Ru | 1.421 | 4.99 | 2100.8  μg h^−1^ cm^−2^ | -- | [17] |
| NiCoBDC@HsGDY | 1.47 | 3.66 | 66.2  μmol h^–1^ cm^–2^ | -- | [18] |
| Ag@Cu_3_N/CF | 1.081 | 12.08 | -- | 30 | [19] |
| Mo_1_Fe_1_Pd | 1.477 | 13.4 | 6  μmol h^–1^ cm^–2^ | 45 | [20] |
| Cu₂O/Cu(OH)_2_@Ni(OH)_2_ | 1.45 | 6.47 | 2.78  mg h^–1^ cm^–2^ | 24 | [21] |
| Ru_3%_-HEO | 0.9 | 9.6 | 1.11  mg h^–1^ cm^–2^ | 72 | [22] |
| NF/Ni_3_N-Cu | 1.45 | 6.28 | 0.088  mmol h^–1^ cm^–2^ | 24 | [23] |
| Cu_3_(HITP)_2_/CF | 1.26 | 7.18 | 0.468  mg h^–1^ cm^–2^ | 18 | [24] |
| Fe-Fe_x_Ni_2-x_P/CeO_2_ | 1.31 | 20.5 | 1.9  mg h^–1^ cm^–2^ | 16 | [25] |

**References**

[1] B. Ravel, M. Newville, “Athena, Artemis, Hephaestus: Data Analysis for X-Ray Absorption Spectroscopy Using Ifeffit”, *Journal of Synchrotron Radiation* 12, no. 4 (2005): 537-541, <https://doi.org/10.1107/s0909049505012719>.

[2] Z. Zhang, Y. Liu, X. Su, Z. Zhao, Z. Mo, C. Wang, Y. Zhao, Y. Chen, S. Gao, “Electro-Triggered Joule Heating Method to Synthesize Single-Phase CuNi Nano-Alloy Catalyst for Efficient Electrocatalytic Nitrate Reduction toward Ammonia”, *Nano Research* 16, no. 5 (2023): 6632-6641, <https://doi.org/10.1007/s12274-023-5402-y>.

[3] Y. Zhao, Y. Liu, Z. Zhang, Z. Mo, C. Wang, S. Gao, “Flower-Like Open-Structured Polycrystalline Copper with Synergistic Multi-Crystal Plane for Efficient Electrocatalytic Reduction of Nitrate to Ammonia”, *Nano Energy* 97, (2022): 107124, <https://doi.org/10.1016/j.nanoen.2022.107124>.

[4] J. P. Perdew, K. Burke, M. Ernzerhof, “Generalized Gradient Approximation Made Simple”, *Physical review letters* 77, no. 18 (1996): 3865, <https://doi.org/10.1103/physrevlett.77.3865>.

[5] X. Jin, L. Sun, X. Wang, R. Wang, L. Yan, A. C. Fisher, J. M. Lee, X. Wang, “Localized Active H* Enrichment by Cobalt Molecular Catalysts for Enhanced Electrocatalytic Nitrate Reduction”, *Angewandte Chemie International Edition* 65, no. 8 (2026): e24566, <https://doi.org/10.1002/anie.202524566>.

[6] Y. Xu, Y. Wen, T. Ren, H. Yu, K. Deng, Z. Wang, X. Li, L. Wang, H. Wang, “Engineering the Surface Chemical Microenvironment over CuO Nanowire Arrays by Polyaniline Modification for Efficient Ammonia Electrosynthesis from Nitrate”, *Applied Catalysis B: Environment and Energy* 320, (2023): 121981, <https://doi.org/https://doi.org/10.1016/j.apcatb.2022.121981>.

[7] S. Luo, H. Guo, T. Li, H. Wu, F. Zhang, C. Tang, G. Chen, G. Yang, Y. Zhou, “Ruthenium-Induced Hydrolysis Effect on Fe_2_O_3_ Nanoarrays for High-Performance Electrochemical Nitrate Reduction to Ammonia”, *Applied Catalysis B: Environment and Energy* 351, (2024): 123967, <https://doi.org/10.1016/j.apcatb.2024.123967>.

[8] L. Qiao, D. Liu, A. Zhu, J. Feng, P. Zhou, C. Liu, K. W. Ng, H. Pan, “Nickel-Facilitated in-Situ Surface Reconstruction on Spinel Co_3_O_4_ for Enhanced Electrochemical Nitrate Reduction to Ammonia”, *Applied Catalysis B: Environment and Energy* 340, (2024): 123219, <https://doi.org/10.1016/j.apcatb.2023.123219>.

[9] Y. Ren, S. You, Y. Wang, J. Yang, Y. Liu, “Bioinspired Tandem Electrode for Selective Electrocatalytic Synthesis of Ammonia from Aqueous Nitrate”, *Environmental Science & Technology* 58, no. 4 (2024): 2144-2152, <https://doi.org/10.1021/acs.est.3c09759>.

[10] Y. Yang, Y. Sun, Y. Wang, X. Zhang, W. Zhang, Z.-F. Huang, L. Yin, A. Han, G. Liu, “Self-Triggering a Locally Alkaline Microenvironment of Co_4_Fe_6_ for Highly Efficient Neutral Ammonia Electrosynthesis”, *Journal of the American Chemical Society* 147, no. 10 (2025): 8893-8905, <https://doi.org/10.1021/jacs.5c00688>.

[11] W. Tahir, Y. Wei, M. Wang, I. E. Khalil, P. Das, T. Wang, C. Cheng, S. Li, A. Thomas, “Covalent Organic Frameworks on Cu_2_O Nanocubes as Rapid Proton/Electron Transfer Gates for Efficient Nh_3_ Electrosynthesis from Nitrate in Neutral Media”, *Journal of the American Chemical Society* 148, no. 1 (2026): 743-755, <https://doi.org/10.1021/jacs.5c16080>.

[12] X. Jia, Y. Kong, D. Wan, L. Liu, S. He, X. Liu, H. Yang, Q. Hu, X. Zhang, C. He, “Self-Sustaining Dynamic Alkaline Microenvironment-Mediated Efficient Nitrate Electroreduction to Ammonia on Mnfeox in Neutral Electrolyte”, *Angewandte Chemie International Edition*, no. (2026): e4598330, <https://doi.org/10.1002/anie.4598330>.

[13] Y. Xu, C. Cheng, J. Zhu, B. Zhang, Y. Wang, Y. Yu, “Sulphur-Boosted Active Hydrogen on Copper for Enhanced Electrocatalytic Nitrate-to-Ammonia Selectivity”, *Angewandte Chemie International Edition* 63, no. 16 (2024): e202400289, <https://doi.org/10.1002/anie.202400289>.

[14] Y. Liu, S. Huang, J. Lu, S. Niu, P. K. Shen, Z. Hu, P. Tsiakaras, S. Gao, “Ni_0.25_cu_0.5_sn0_.25_ Nanometallic Glasses as Highly Efficient Catalyst for Electrochemical Nitrate Reduction to Ammonia”, *Advanced Functional Materials* 34, no. 52 (2024): 2411325, <https://doi.org/10.1002/adfm.202411325>.

[15] Y. Liu, J. Ma, S. Huang, S. Niu, S. Gao, “Highly Dispersed Copper-Iron Nanoalloy Enhanced Electrocatalytic Reduction Coupled with Plasma Oxidation for Ammonia Synthesis from Ubiquitous Air and Water”, *Nano Energy* 117, (2023): 108840, <https://doi.org/10.1016/j.nanoen.2023.108840>.

[16] H. Lin, J. Wei, Y. Guo, Y. Li, X. Lu, C. Zhou, S. Liu, Y. y. Li, “Bi_1_-CuCo_2_O_4_ Hollow Carbon Nanofibers Boosts NH_3_ Production from Electrocatalytic Nitrate Reduction”, *Advanced Functional Materials* 34, no. 51 (2024): 2409696, <https://doi.org/10.1002/adfm.202409696>.

[17] Y. Yao, X. Wei, H. Zhou, K. Wei, B. Kui, F. Wu, L. Chen, W. Wang, F. Dai, P. Gao, N. Wang, W. Ye, “Regulating the d-Band Center of Metal-Organic Frameworks for Efficient Nitrate Reduction Reaction and Zinc-Nitrate Battery”, *ACS Catalysis* 14, no. 21 (2024): 16205-16213, <https://doi.org/10.1021/acscatal.4c04340>.

[18] J. Ma, Y. Zhang, B. Wang, Z. Jiang, Q. Zhang, S. Zhuo, “Interfacial Engineering of Bimetallic Ni/Co-MOFs with H-Substituted Graphdiyne for Ammonia Electrosynthesis from Nitrate”, *ACS Nano* 17, no. 7 (2023): 6687-6697, <https://doi.org/10.1021/acsnano.2c12491>.

[19] H. Dong, J. Ye, X. Liu, H. Zhang, L. Yuan, W. Tian, J. Ji, “Spatially Separated Ag@Cu_3_N Tandem Electrocatalyst with High Nitrate-to-Ammonia Selectivity via Decoupled Deoxygenation‐Hydrogenation Pathway”, *Advanced Functional Materials*, no. (2025): e26882, <https://doi.org/10.1002/adfm.202526882>.

[20] W. Ye, Y. Yao, X. Wei, M. Xu, S. Zhao, W. Wang, G. Jia, F. Dai, P. Gao, X. Lu, X. Li, B. Xi, N. Wang, S. Xiong, “Continuous Intermediates Spillover Boosts Electrochemical Nitrate Conversion to Ammonia over Dual Single‐Atom Alloy”, *Angewandte Chemie International Edition* 64, no. 32 (2025): e202509303, <https://doi.org/10.1002/anie.202509303>.

[21] T. Lv, L. Yang, C. Hong, Y. Zhu, J. Shen, C. Li, “Electronic Interaction at Cu-O-Ni Heterointerface Promotes Electrocatalytic Nitrate Reduction to Ammonia and Zinc-Nitrate Battery”, *Advanced Science* 13, no. 14 (2026): e21252, <https://doi.org/10.1002/advs.202521252>.

[22] H. Guo, Z. Guo, G. Xue, H. Wang, J. Gong, K. Chu, J. Qin, Y. Guan, H. Dong, Y. Chen, Y. E. Miao, C. Zhang, H. Liu, T. Liu, J. Hofkens, F. Lai, “Entropy‐Driven Stabilization of Noble Metal Single Atoms: Advancing Ammonia Synthesis and Energy Output in Zinc-Nitrate Batteries”, *Advanced Materials* 37, no. 28 (2025): 2500224, <https://doi.org/10.1002/adma.202500224>.

[23] X. Ouyang, W. Qiao, Y. Yang, B. Xi, Y. Yu, Y. Wu, J. Fang, P. Li, S. Xiong, “Intensifying Interfacial Reverse Hydrogen Spillover for Boosted Electrocatalytic Nitrate Reduction to Ammonia”, *Angewandte Chemie International Edition* 64, no. 13 (2025): e202422585, <https://doi.org/10.1002/anie.202422585>.

[24] W. Wang, B. Chen, J. Guo, Z. Liu, H. Yuan, B. Fan, X. Huang, “Fabrication of a Novel Cu Based Conjugated Coordination Polymer for Effective Electroreduction of Nitrate to Ammonia and Zn-Nitrate Batteries”, *Advanced Functional Materials* 35, no. 31 (2025): 2501057, <https://doi.org/10.1002/adfm.202501057>.

[25] X. Xie, Y. Zhong, P. Muthukumar, B. Zhang, J. Yang, J. K. Sun, X. Yang, H. M. Cheng, “Enzyme‐Mimicking Metal–Phosphide Tandem Catalytic Centers for Efficient Electrochemical Nitrate‐to‐Ammonia Conversion and Zinc-Nitrate Battery”, *Angewandte Chemie International Edition* 65, no. 10 (2026), <https://doi.org/10.1002/anie.202525416>.
